# Phage Display against Corneal Epithelial Cells Produced Bioactive Peptides That Inhibit *Aspergillus* Adhesion to the Corneas

**DOI:** 10.1371/journal.pone.0033578

**Published:** 2012-03-12

**Authors:** Ge Zhao, Siyuan Li, Wei Zhao, Kun He, Haijie Xi, Weihua Li, Qingjun Zhou, Yiqiang Wang

**Affiliations:** 1 Shandong Provincial Key Laboratory of Ophthalmology, Shandong Eye Institute, Shandong Academy of Medical Sciences, Qingdao, China; 2 Department of Microbiology, Qingdao University Medical College, Qingdao, China; 3 Institute of Basic Medical Sciences, National Center of Biomedical Analysis, Beijing, China; Duke University Medical Center, United States of America

## Abstract

Dissection of host-pathogen interactions is important for both understanding the pathogenesis of infectious diseases and developing therapeutics for the infectious diseases like various infectious keratitis. To enhance the knowledge about pathogenesis infectious keratitis, a random 12-mer peptide phage display library was screened against cultured human corneal epithelial cells (HCEC). Fourteen sequences were obtained and BLASTp analysis showed that most of their homologue counterparts in GenBank were for defined or putative proteins in various pathogens. Based on known or predicted functions of the homologue proteins, ten synthetic peptides (Pc-A to Pc-J) were measured for their affinity to bind cells and their potential efficacy to interfere with pathogen adhesion to the cells. Besides binding to HCEC, most of them also bound to human corneal stromal cells and umbilical endothelial cells to different extents. When added to HCEC culture, the peptides induced expression of MyD88 and IL-17 in HCEC, and the stimulated cell culture medium showed fungicidal potency to various extents. While peptides Pc-C and Pc-E inhibited *Aspergillus fumigatus (A.f)* adhesion to HCEC in a dose-dependent manner, the similar inhibition ability of peptides Pc-A and Pc-B required presence of their homologue ligand Alb1p on *A.f.* When utilized in an eyeball organ culture model and an *in vivo A.f* keratitis model established in mouse, Pc-C and Pc-E inhibited fungal adhesion to corneas, hence decreased corneal disruption caused by inflammatory infiltration. Affinity pull-down of HCEC membrane proteins with peptide Pc-C revealed several molecules as potential receptors for this peptide. In conclusion, besides proving that phage display-selected peptides could be utilized to interfere with adhesion of pathogens to host cells, hence could be exploited for managing infectious diseases including infectious keratitis, we also proposed that the phage display technique and the resultant peptides could be used to explore host-pathogen interactions at molecular levels.

## Introduction

Infectious keratitis (IKs) is a large group of vision-threatening diseases caused by infections of corneas with various pathogens like bacteria, fungi, acanthamoeba, virus, and multi-cell parasites such as *onchocerca volvulus*. If not controlled properly, IKs can lead to the loss of sight in the infected eye, or enucleation is required for controlling infection [1]. The spectrum of pathogens causing IKs varies with time and geometry [2], but fungal keratitis (FK) dominates among hospitalized IK patients in developing countries like China [3], [4]. Compared to the well-formed studies involving viral or bacterial keratitis, the pathogenesis of FK is less clear and much of the current knowledge about the mechanisms of FK is simply adopted from studies on fungal infection in other tissues [5]. For most tissues with open surfaces accessible to microbes, adhesion of microbes to the epithelial or endothelial cells is usually the first step for establishment of a commensal or a pathogenic relationship [6], [7]; this might be mediated by the binding of pathogen ligands to host receptors. This initial adhesion usually activates or changes the status of both host cells and pathogens, leading to cross-talk in the form of either cellular surface ligand-receptor coupling or secretion of soluble mediators. Often several ligand-receptor pairs or communication types are involved in the host-pathogen interactions, and result in removal of pathogens, sometimes accompanied with destructive outcome in the affected tissues. Theoretically, interfering with the ligand-receptor coupling by the simulation of ligands or receptors might block pathogen invasion, and thus serves as a good strategy for prevention or treatment of infection especially in the early stage. Some host extracellular matrix components like types I and IV collagens, fibronectin, and basement membrane laminin have been proposed to mediate host-pathogen binding, but the molecules on the pathogen surface have still to be identified [8]–[11]. Furthermore, in the case of FK, the molecules on the corneas that are bound by pathogens are unclear as well.

On the other hand, phage display (PhD) [12]–[14] has been proven to be powerful for studying protein-protein or protein-tissue interactions [15]. In the area of host-pathogen interaction, PhD has been successfully used for discovering new pathogen ligands that bind host receptors during the adhesion stage [16]–[18]. For example, the PhD peptide library was widely used for determining the functional cell-specific binding motifs of mammal cells [19]–[21] and pathogens [18], [22], and a 23-mer peptide containing the cell binding domain effectively inhibited the adherence of *Candida albicans* to extracellular matrix proteins [23]. In an attempt to dissect the mechanisms of IK, we used PhD to screen for peptides that bind human corneal epithelial cells (HCEC). Theoretically, these peptides could be used in two ways. First their sequences could be used for identifying potential pathogen ligands that are homologue to these peptides thus also bind HCEC. Second, these peptides, together with their homologue ligands, could be used for identifying host receptors. The peptides might also be used for translational purposes, such as blocking the adhesion of corresponding pathogens to the host and, in situations that binding of peptides or putative pathogen ligands to host cells induces protective responses, the corresponding peptides might also work as substitutes to induce this protective effect. Hence, we studied the effect of the resultant peptides using pathogen adhesion models at *in vitro, ex vivo* and *in vivo* levels respectively, and proved the feasibility and usefulness of this strategy.

## Results

### PhD selected peptide sequences and BLAST analysis

**Figure 1:**
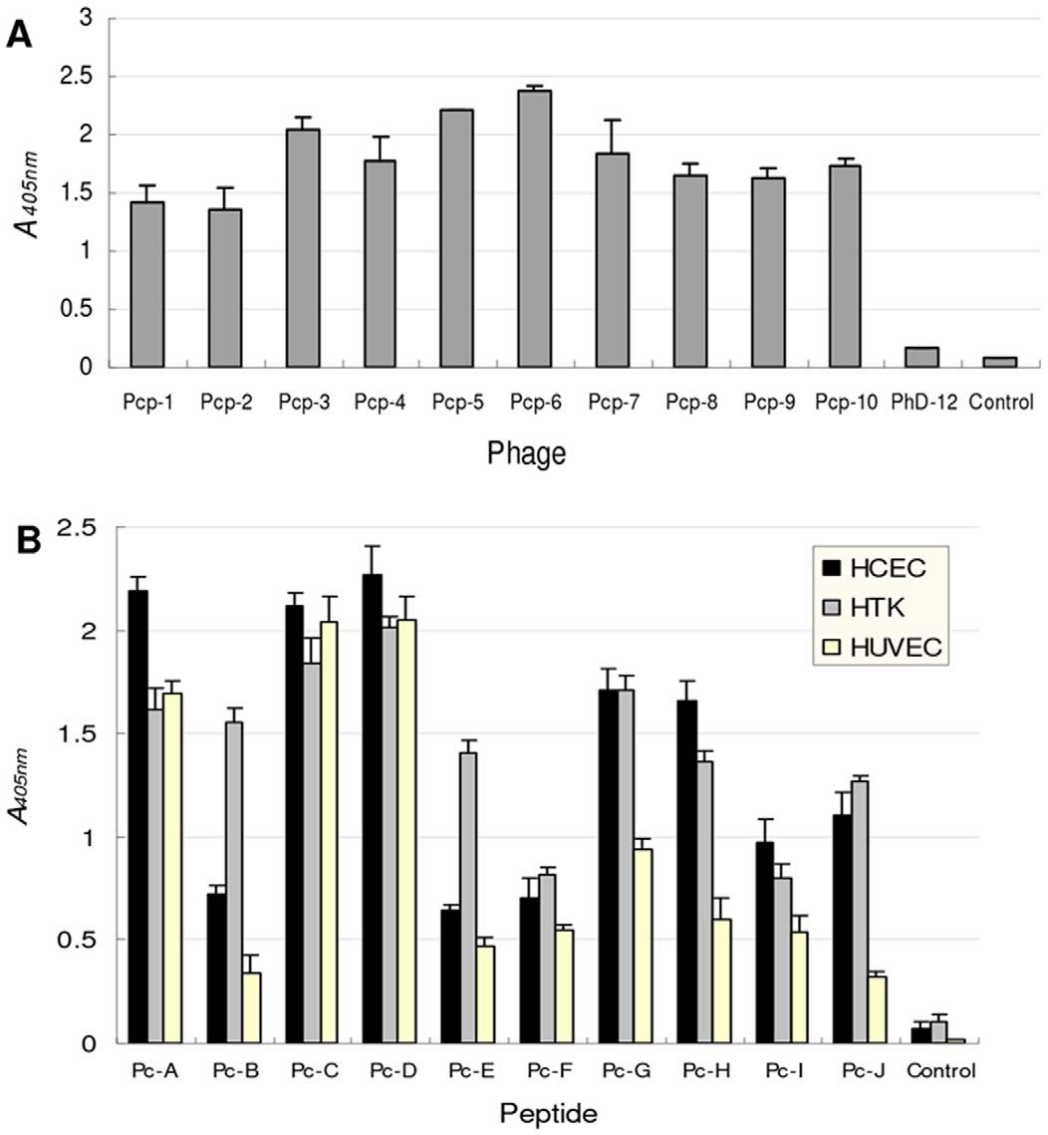
Selected monoclonal phages or synthesized peptides show increased affinity for cells. (A) Representatives of the selected monoclonal phages (Pcp-1 to Pcp-10) showed increased affinity for HCEC over starting mixed phage library (PhD-12). For ELISA measurement of binding affinity of phages, confluent monolayer HCEC were incubated with 10^12^ PFU amplified phages for 1 hour after blocking with BSA. HRP-conjugated anti-M13 antibody was added for another 1 hour, and then tetramethyl benzidine was added for coloration. (B) Adhesion of representative peptides to cells depends on peptide sequence and cell types. The affinity of each of the 100 µM synthesized peptides for binding cells, including HCEC, HTK and HUVEC, is different by ELISA assay. Culture medium without any peptides was used as control.

After three rounds of bio-panning starting from the 12-mer PhD peptide library against cultured HCEC lines, 40 phage infected bacteria clones were randomly selected for sequencing, and 14 different DNA sequences were identified (Table 1, Table S1). Each of the selected phages showed increased affinity for HCEC as confirmed with the ELISA method (Fig. 1A). Homology analysis of the corresponding 12-mer peptide sequences peptide with GenBank data was carried out using the online NCBI BLASTP program without limiting the species resources. Surprisingly, the dominant majority of the homologue sequences retrieved for each peptide was for proteins in various pathogens like fungi, bacteria, and to lesser extent, virus (Table S2). Very few of the returning homologue proteins were from vertebrate species. Among these homologues, some were well-defined proteins with formal nomenclature, while others were classified only as hypothetical or putative proteins. When the top 100 homologues with the highest alignment scores in *Aspergullus fumigatus (A.f)* for each of the 14 peptides were combined and subjected to annotation and clustering analysis with the Database for the Annotation, Visualization, and Integrated Discovery (DAVID, v6.7) program, most of the genes are involved in metabolism or meiosis pathways (Table S3). Some representatives of the homologue sequences in *A.f* proteins relating to these 14 peptides are listed in Table 1. It is noteworthy that Pc-A and Pc-B were homologue to different parts of polyketide synthase Alb1p (ACJ13039), an enzyme important in determining the virulence of *A.f* [24], [25]. Since *A.f* is among the leading causes of FK in many parts of the world, we focused our following experimental studies on this model pathogen. Based on the judgment on the potential roles of homologue proteins in *A.f*, ten peptides (Pc-A to Pc-J) were synthesized for functional studies.

**Table 1:** Peptide sequences that bind HCEC and show homologue to *A. fumigatus* proteins.

Peptide code and sequence	Description of homologue *Aspergillus* fumigatus sequence of interest
Pc-A: ATKVKIPFEAKV	ACJ13039: polyketide synthase Alb1p
(CACCTTCGCCTCAAAAGGAATCTTCACCTTAGTAGC)	EDP49269 :NACHT and Ankyrin domain protein
	EDP47502 :HATPase_c domain protein, putative
	EDP50664: glutamine synthetase
Pc-B: VATPVPPTLTPF	ACJ13039: polyketide synthase Alb1p
(AATCGGAGTCAGAGTCGGCGGAACCGGCGTCGCAAC)	XP_749784: hypothetical protein AFUA_1G00320
	EDP47447: sodium transporting ATPase, putative
Pc-C: ATLRTYPYMDRA	XP_749787: cell surface metalloreductase, putative
(AGCCCGATCCATATAAGGATACGTACGCAGCGTAGC)	XP_750165: aspartate aminotransferase, putative
	EDP54828: bifunctional tryptophan synthase TRPB
Pc-D: QLAPMATHDKHP	XP_746369: antigenic cell wall galactomannoprotein, putative
(CGGATGCTTATCATGAGTAGCCATCGGAGCAAGCTG)	XP_751254: MFS multidrug transporter
Pc-E: YALRPGMPQWLE	XP_750173: endo-1,3-beta-glucanase Engl1
(AGCACGCGTAGGATTAGGAAACGGCGACTCCGCATG)	EDP50333: ZIP Zinc transporter, putative
	EDP53374: MFS multidrug transporter, putative
	XP_747279: oligopeptidase family protein
Pc-F: TPPTYSWFTHRM	XP_751660: Leucine rich repeat domain protein
(CTCAAGCCACTGCGGCATACCAGGCCTCAACGCATA)	XP_746913: Putative polyketide synthase
	XP_754859: exo-beta-1,3-glucanase, putative
Pc-G: GSATNPTMGQRM	EDP54833: hypothetical protein AFUB_028930
(CGCAATAGACGAATTCGAATGCAAAGTAATCTTATT)	XP_001481671: GYF domain protein
	XP_747950: alpha-1,3-glucanase
Pc-H: AETHVLNKHTPL	XP_752425: GPI anchored protein, putative
(ACGCCGCCTACGTATTCTTGGTTTACTCATCGTATG)	XP_747678: GAS2 domain protein
Pc-I: HSSSHWSWSTPL	XP_753005: MFS hexose transporter, putative
(GGTTCGGCTACTAATCCGACGATGGGTCAGCGGATG)	XP_750940: 3-oxoacyl-acyl carrier protein reductase
Pc-J: NMRLLANPAMAG	XP_747167: polyketide synthase
(GCTGAGACGCATGTTCTGAATAAGCATACTCCGCTG)	XP_752055: clathrin heavy chain
Pc-K: QIPAQNRLVFLT	XP_750702: checkpoint protein kinase (SldA), putative
(CGTCAAAAACACCAGACGATTCTGCGCAGGAATCTG)	XP_749268: Potassium/sodium P-type ATPase
Pc-L: VPGWDSHNARHQ	XP_746960: MFS transporter, putative
(CTAATGCCGCGCATTATGACTATCCCAACCAGGCAC)	
Pc-M: HAESPFPNPTRA	XP_749029: Conserved hypothetical protein
(AGCACGCGTAGGATTAGGAAACGGCGACTCCGCATG)	XP_746882: ABC multidrug transporter, putative
Pc-N: NKITLHSNSSIA	XP_749340: peptidyl-prolyl cis-trans isomerase Cpr7
(CGCAATAGACGAATTCGAATGCAAAGTAATCTTATT)	XP_755784: conserved hypothetical protein

### Adhesion of peptides to cells depends on peptide sequence and cell types

Whole-cell ELISA was first performed to assess the binding affinity and specificity of the synthetic peptides to HCEC and two other cell lines, namely human corneal stromal fibroblast cells (HTK cell line) and human umbilical vascular endothelial cells (HUVEC). Besides confirming the different affinity for HCEC, results showed that the peptides also bound HTK and HUVEC to various extents (Fig. 1B). For example, while Pc-C and Pc-D produced similar binding to all three cells respectively, Pc-B and Pc-E showed significantly higher binding to HTK than to HCEC or HUVEC. These facts implied that the actual binding of the peptides depended not only on peptide sequences, but also on the type of the target cells.

### Adhesion of peptides to HCEC activates cytokines production

**Figure 2:**
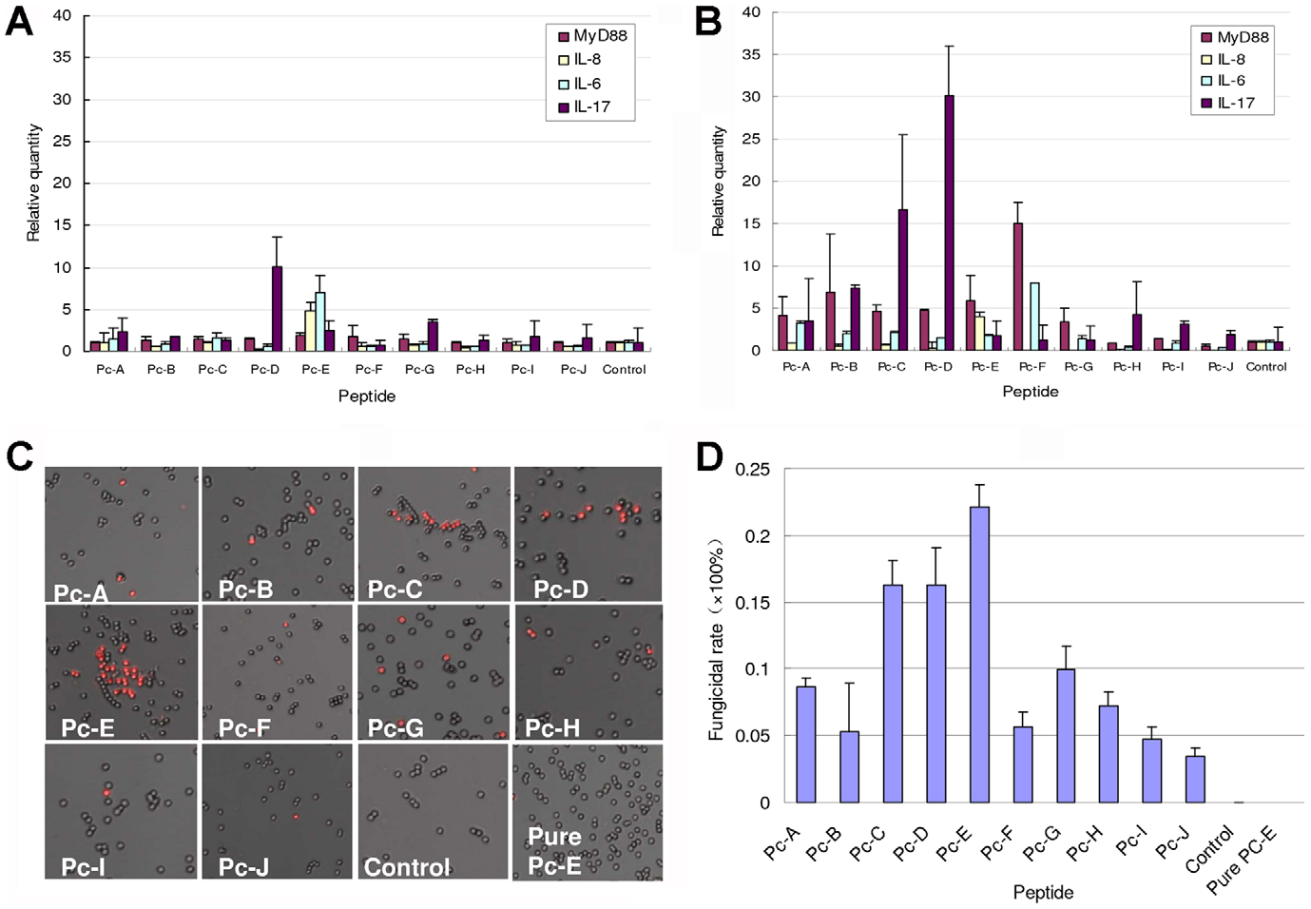
Adhesions of peptides to HCEC activate production of cytokines and fungicidal factors. (A–B) Cytokines expression is changed in HCEC stimulated by different peptides for 1 hour (A) and 4 hours (B). After HCEC incubated with 100 µM peptides for 1 hour, Pc-D stimulated the highest IL-17 production and Pc-E stimulated the most IL-6 and IL-8 production. After 4 hours incubation, IL-17 production stimulated by Pc-B, Pc-C, and Pc-D was much higher, and production of MyD88, stimulated by Pc-E, was obviously increased in 7 peptides. (C–D) Measurement of the effect of supernatant obtained from peptides-treated HCEC culture on survival of *A.f.* 5×10^5^ CFU conidia were seeded into 96 well plates and the supernatants obtained from HCEC, stimulated with 100 µM peptides for 1 hour, were added for 2 hours incubation at 33°C. Dead cells were detected under confocal microscope after PI staining (C), and the fungicidal rate of each peptide to *A.f* was calculated (D). Supernatant from Pc-E-stimulated HCEC displayed the highest fungicidal activity on *A.f*, but medium contain the pure Pc-E had no effect on conidia survival.

Homology with pathogen proteins involved in host-pathogen interaction might award the peptides ability to stimulate HCEC via their putative receptors on cells. To track whether HCEC initiated any protective or inflammatory response upon encountering peptides, the toll-like receptor-signaling pathway adaptor MyD88 (NM_002468) and the inflammatory cytokines IL-6 (NM_000600), IL-8 (NM_000584), IL-17 (NM_002190) were measured using real-time PCR for their expression in HCEC. Fig. 2 shows that the cells responded to peptide treatment differentially. In detail, one hour after treatment with peptides, Pc-D stimulated the highest IL-17 production and Pc-E stimulated the most IL-6 and IL-8 production among all peptides (Fig. 2A). By four hours of treatment, the production of cytokines stimulated by Pc-E did not appreciatively change, but IL-17 production stimulated by Pc-B, Pc-C, and Pc-D was much higher (Fig. 2B).

The study also tested whether the peptide-stimulated HCEC produced any fungicidal factors into the medium. By measuring the survival or growth of *A.f in vitro*, it was shown that the supernatants obtained from Pc-E stimulation displayed the highest fungicidal activity on *A.f* conidia (Fig. 2C, D), followed by Pc-C, Pc-D, Pc-G, Pc-A and Pc-H respectively. The culture medium containing only peptides without cells had no effect on fungal growth (Fig. 2C, D, and data not shown). It is noteworthy that the relative efficacy of each peptide to activate HCEC depends on the readouts of interest. For example, both Pc-C and Pc-D induced more upregulation of IL-17 production versus IL-6, but Pc-G induced more IL-6 versus IL-17. Together with the complexity of the induced antifungal effect of different peptides, these data implied that the peptides may activate HCEC via different receptors.

### Peptides inhibited *A. fumigatus* adherence to HCEC

**Figure 3:**
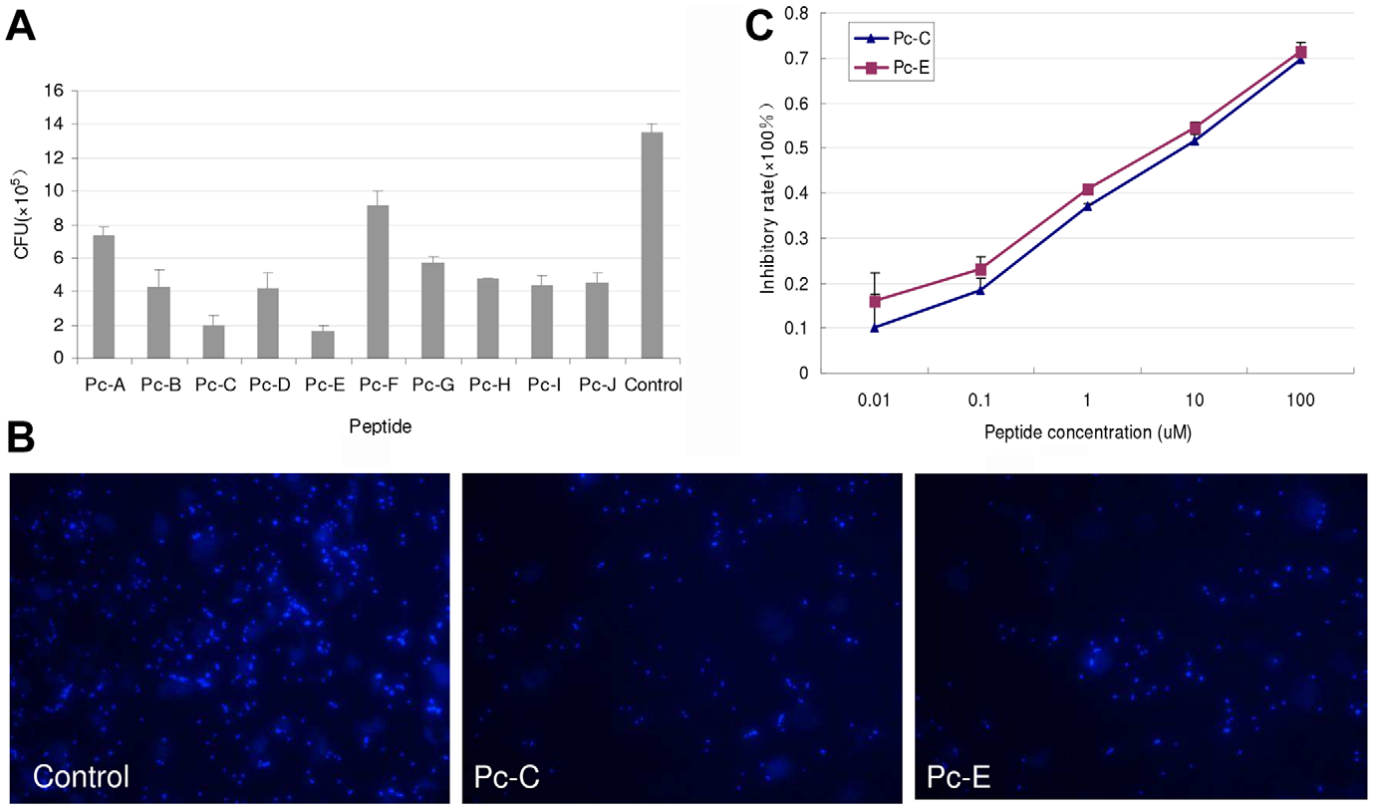
Peptides display inhibitory effect on *Aspergillus* adherence to HCEC. (A) Pretreatment of HCEC with 10 peptides inhibit *A.f* adherence to HCEC to different extents. HCEC were incubated with 500 µL of 100 µM screened peptides for 1 hour, followed by inoculation with 10^7^ CFU *A.f* conidia for another 1 hour at 37°C. The cells were lysed and spread on plates for 48 hours of culture; then the number of colonies was counted. (B) Pc-C and Pc-E inhibited conidia of *A.f* adherence to HCEC. The adhered fungal conidia were directly detected under a confocal microscope after staining with Calcofluor White for 5 min. (C) Pc-C and Pc-E inhibited *A.f* adherence to HCEC. Different concentrations (0, 0.01, 0.1, 1, 10, and 100 µM) of peptide Pc-C and Pc-E were used for inhibitory assay of *A.f* adherence to HCEC. Medium containing not any peptides was used control.

Since the pathogen proteins homologue to the peptides might participate in host-pathogen interactions, we next studied if the PhD peptides interfere with fungal adherence to HCEC. Fig. 3A shows that pretreatment of HCEC with peptides inhibited *A.f* adherence to HCEC to different extents. The inhibitory effects of Pc-C and Pc-E were especially prominent, either by CFU assay or by direct observation under a microscope of the fungi attached to HCEC (Fig. 3B). Moreover, the inhibition of *A.f* adherence by Pc-C and Pc-E showed a dose-effect relationship, with the IC50 of Pc-C and Pc-E being about 4.79 µM and 3.02 µM respectively (Fig. 3C). So we chose Pc-C and Pc-E for following studies.

### Low cytotoxicity of peptides Pc-C and Pc-E on HCEC culture

**Figure 4:**
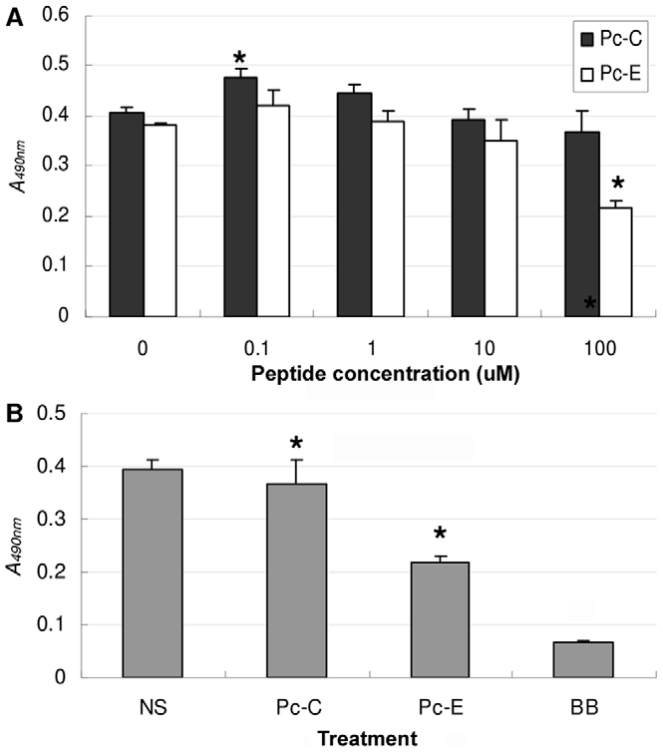
Cytotoxicity of Pc-C and Pc-E to HCEC by MTT assay. (A) No cytotoxicity of Pc-C to HCEC was detected at any concentration; 0.1 µM Pc-C slightly but significantly enhanced HCEC proliferation (*P*<0.05), while significant cytotoxicity of Pc-E was detected at a concentration of 100 µM (*P*<0.05). HCEC were incubated in medium with peptide (0, 0.01, 0.1, 1, 10, 100 µM) for 72 hours, followed by 4 hours incubation with MTT. (B) The cytotoxicity of 100 µM Pc-E to HCEC was significantly lower than that of 0.01% Benzalkonium Bromide (BB) (*P*<0.05).

To detect whether the peptides were safe for potential therapeutic use like in FK management, we measured the effect of Pc-C and Pc-E on HCEC survival. The activation of HCEC upon peptide stimulation did not cause obvious change in the appearance or growing pattern of the cells. MTT assay of the cells showed that peptide Pc-C up to 100 µM did not show any cytotoxicity to HCEC (Fig. 4A). On the contrary, 0.1 µM Pc-C slightly enhanced HCEC proliferation (*P* = 0.003). With Pc-E, however, significant cytotoxicity was detected at a concentration of 100 µM (*P*<0.001), but not at lower concentrations. For comparison, Fig. 4B shows that the cytotoxicity of 100 µM Pc-E to HCEC culture was significantly lower than that of 0.01% Benzalkonium Bromide (*P*<0.001), a usual concentration of this common preservative used in various eye drops. But more extensive studies will be necessary before a conclusion could be reached concerning the *in vivo* safety or toxicity of these peptides.

### Peptides protected corneas from infection of *A. fumigatus* in *ex vivo* and *in vivo* FK models

**Figure 5:**
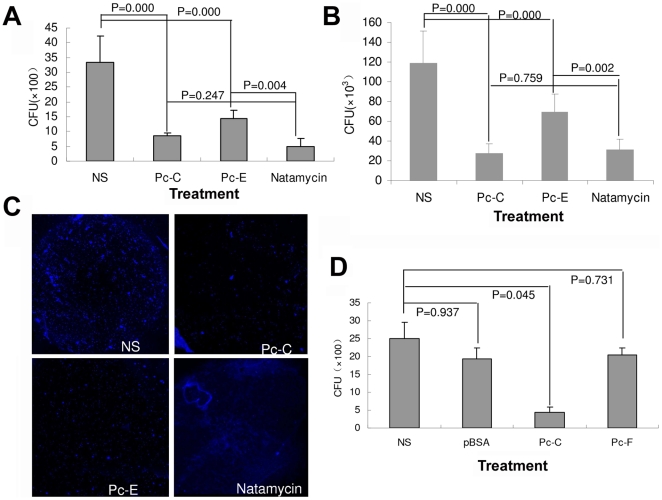
Peptides inhibit *A. fumigatus* adherence to corneas in ex vivo and in vivo. (A–B) Peptide Pc-C and Pc-E significantly inhibited *A.f* conidia adherence to corneas in both eyeball culture (A) and live Balb/c mice (B). In the *ex vivo* model, corneal epithelia were scarified, and in the *in vivo* model, the corneal epithelia were blotted with paper as detailed in the Method section. In both conditions, 100 µM peptide Pc-C or Pc-E was added to the corneal surface for 1 hour, followed by inoculation with 10^7^ CFU *A.f* conidia for another 1 hour. Then the eyes were washed and the corneas were excised and homogenated by ultrasonication. The samples were spread on plates and cultured for 48 hours. The fungal colonies were counted. Pc-C showed stronger inhibitory effects than Pc-E, and the effect of Pc-C was comparable to that of Natamycin Eye Drops. (C) Fungal conidia on the corneal surface in an *in vivo* model were detected by confocal microscope after staining with Calcofluor White. Pc-C and Pc-E decreased fungal adhesion to corneas but Natamycin Eye Drops did not. (D) Other two peptides pBSA and Pc-F were also assayed along with Pc-C in *ex vivo* model, but neither of them displayed any significant inhibitory effect on *A.f* adherence to corneas.

Next, the study examined whether Pc-C and Pc-E interfere with the infection of corneas when exposed to *A.f* at organ levels using *ex vivo* or *in vivo* FK models. Fig. 5 shows that peptide Pc-C and Pc-E significantly inhibited *A.f* conidia adherence to corneas by 2∼3 fold in both excised eyeball culture (*ex vivo*) and live Balb/c mice (*in vivo*). In both models, Pc-C showed stronger inhibitory effects than Pc-E, and the effect of Pc-C at the applied dosage was comparable to that of 5% Natamycin Eye Drops (with benzalkonium chloride 0.02%), an antifungal chemical used in clinical practice. Confocal microscope scanning of whole corneas showed that Pc-C and Pc-E decreased fungal adhesion to corneas but that Natamycin Eye Drops did not (Fig. 5C), confirming that peptides and Natamycin inhibit infection via different mechanisms. In the *ex vivo* FK model, other two peptides pBSA and Pc-F were also assayed along with Pc-C, and they displayed no significant inhibitory effect on *A.f* adherence to corneas (Figure 5 D).

**Figure 6:**
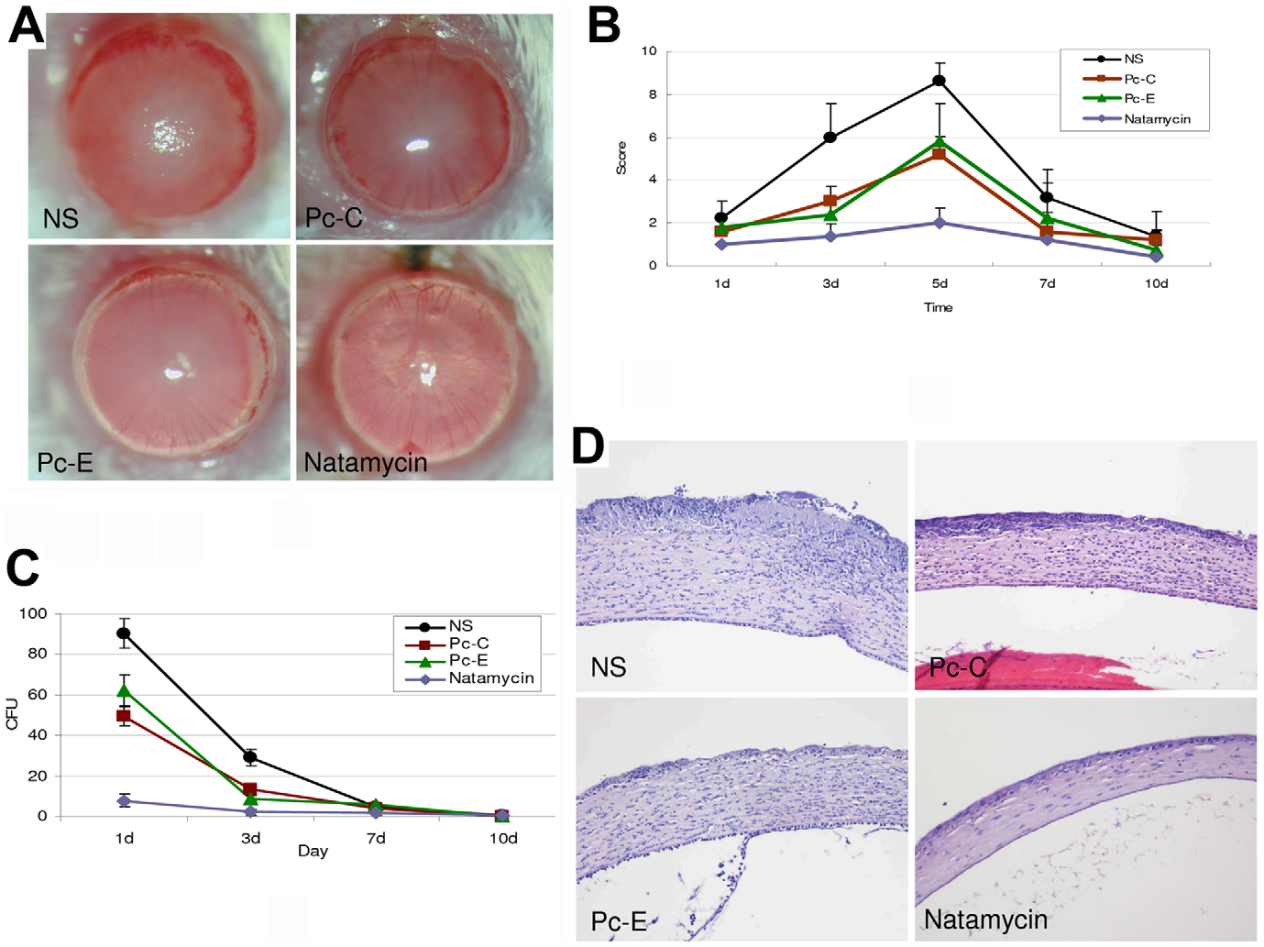
Peptides protect corneas from infection of *A. fumigatus* in vivo. (A–B) Treatment of corneas with peptide Pc-C or Pc-E around infection significantly decreased the disease scores at day 3 and 5 of post infection when compared with mock treated eyes (*P*<0.05), but to less extent than Natamycin. The corneas of Balb/c mice were scarified and they received a 5-µL drop of 100 µM peptide 4 times in 1 hour preinfection. Then the eyes were topically inoculated with 10^7^ CFU *A.f* conidia. After 1 hour of infection, the application of peptide continued hourly for 5 hours. On days 1, 3, 7, 10 and 14, post infection, the development of FK, if any, was monitored with a slit lamp microscope. (C) The quantities of *A.f* recovered at day 1 and 3 post infection from the corneas treated with peptide Pc-C or Pc-E were significantly less than those recovered from the mock treated control but higher than those of Natamycin treated corneas. (D) The inflammatory cell infiltration and edema in Pc-C or Pc-E peptide-treated corneas were much less than in mock-treated corneas, as determined by histopathologic studies at 3 days post infection. Corneas treated with Natamycin did not show obvious histological alteration.

To study whether the blockade of *A.f* adherence to corneal cells could decrease disease development, the peptides were applied to the scratched and inoculated corneas of Balb/c mice, and it was found that treatment of corneas with peptide Pc-C or Pc-E around infection significantly decreased the disease scores at day 3 and day 5 post infection when compared with mock treated eyes (Fig. 6A, B). However, neither of the two peptides reached the high therapeutic effects of Natamycin. For example, at 3 days post infection, dense corneal opacity obscured anterior chamber details in mock treated eyes, while lighter corneal opacity occurred in peptide Pc-C- or Pc-E-treated groups, but the transparency of the corneas was marginally affected in Natamycin treated eyes (Fig. 6A). The quantities of *A.f* recovered from Pc-C- or Pc-E-treated corneas were significantly less than that recovered from the mock-treated control at day 1 and 3 but were higher than that of Natamycin-treated corneas (Fig. 6C). Histopathologic studies at 3 days post infection showed that the inflammatory cell infiltration and edema in Pc-C- or Pc-E-treated corneas were much less than that in mock treated corneas. Corneas treated with Natamycin did not show obvious histological alteration compared with normal corneas (Fig. 6D). Collectively, these findings proved that Pc-C and Pc-E peptides moderately inhibited FK *in vivo* at the studied dosage.

### Combinational use of peptides decreased Natamycin dosage required for inhibition of *A. fumigatus* growth

**Figure 7:**
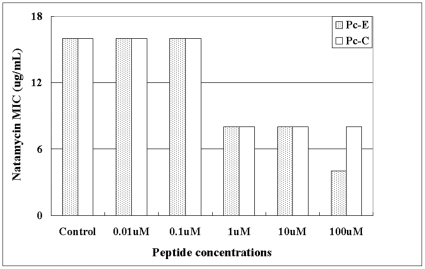
Peptides decrease the minimum inhibitory concentrations of Natamycin required for inhibiting A. fumigatus adhered to HCEC. HCEC grown in 96-well plates were pretreated with peptides Pc-C or Pc-E of different concentrations for 1 hour before 10^6^ CFU *A.f* conidia were added to each well for another 1 hour. After removal of unbound conidia, Natamycin was added to the culture to 2-fold serial concentrations for each peptide concentration, with 2 replicates for each setting. The plates were cultured at 37°C for 48 hours to determine the MIC of Natamycin against the adhered *A.f* conidia.

Since peptides and Natamycin inhibit infection of *A.f* to corneal cells at different stages, supplemental use of peptides with Natamycin might achieve better inhibition or decrease the required dose of Natamycin. Using the *in vitro* minimum inhibitory concentration (MIC) assay in the infection model of HCEC, it was found that 1 µM of peptides Pc-C or Pc-E could effectively decrease the MIC of Natamycin (Fig. 7). Increasing the Pc-E concentration to 100 µM could further decrease Natamycin MIC. The additional or synergistic effect of peptides and Natamycin was not tried in this study.

### Identification of Alb1p as possible pathogen ligand that bind HCEC

**Figure 8:**
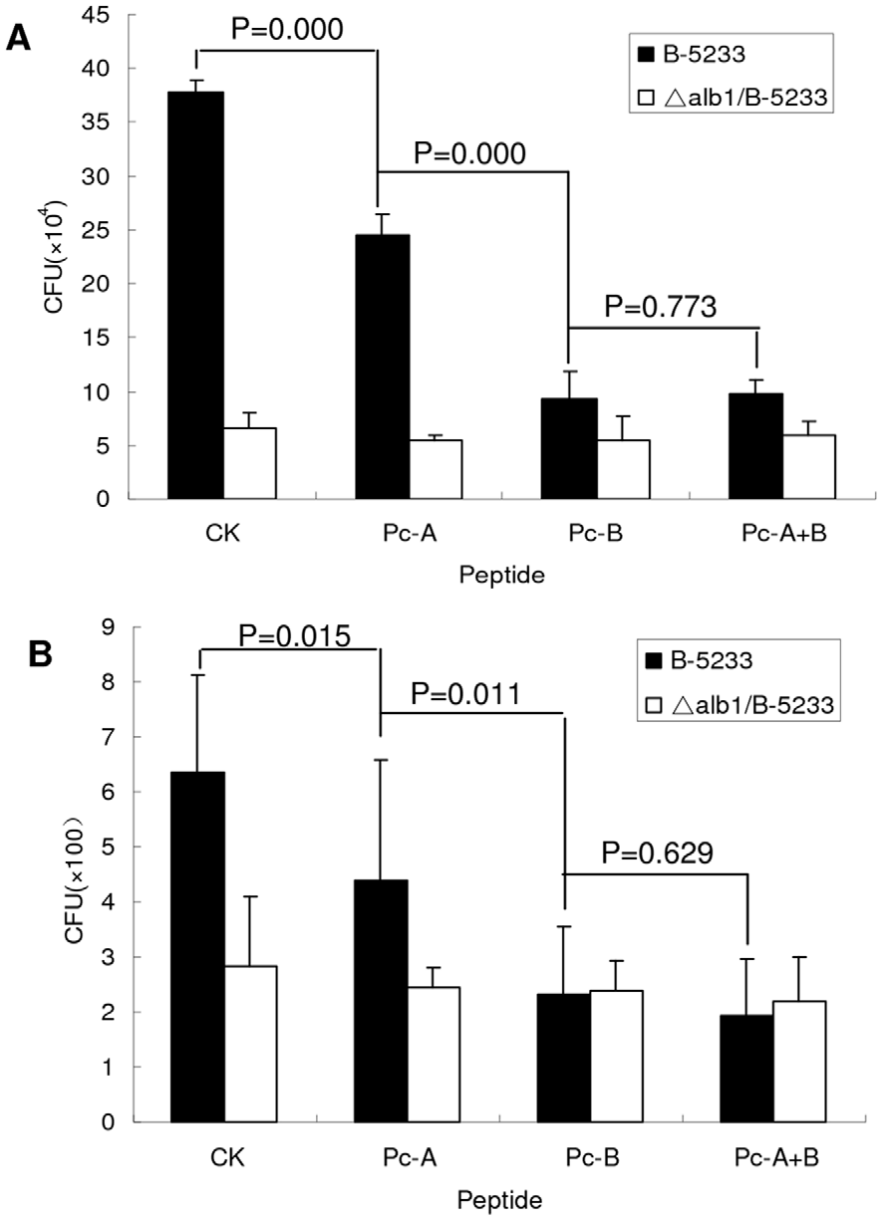
Pc-A and Pc-B inhibit adhesion of *A. fumigatus* but not *Alb1p* mutant to corneal cells. (A–B) Peptides Pc-A and Pc-B significantly inhibited wild-type *A.f* B-5233 adherence to HCEC (A) and corneas of cultured eyeballs (B) (*P*<0.05), but no additive or synergistic effects were observed for combinational use of the two peptides. The adhesion of *Alb1p*-deficient mutant to HCEC and corneal surface were significantly decreased compared with the wild type strain (*P*<0.05). However, neither Pc-A, Pc-B nor their combination could further decrease the adhesion of mutant *A.f* to HCEC or *ex vivo* murine corneas.

To further validate the hypothesis that PhD peptides might be used as the first step for identification of pathogen ligands that responsible for adhesion to host cells, we studied the effects of Pc-A and Pc-B (Table 1) on the binding of wild type or *Alb1p* deficient strains of *A.f* to HCEC. Like Pc-C and Pc-E as demonstrated above, both Pc-A and Pc-B peptides significantly inhibit wild-type *A.f* adherence to HCEC (Fig. 8A) and corneas of cultured eyeballs (Fig. 8B). Just like reported with other adhesion models [24], the adhesion of *Alb1p*-deficient mutant to HCEC and Balb/c corneal epithelium were significantly decreased compared with that of the wild type strain (Fig. 8). However, neither Pc-A, Pc-B nor their combination could further decrease the adhesion of mutant *A.f* to HCEC or *ex vivo* murine corneas, suggesting that Alb1p is required both for wild-type *A.f* to bind corneal cells and for inhibitory effects of its homologue peptides on wide type conidia binding.

### Identification of HCEC membrane proteins interacting with peptide Pc-C

Lastly we tried to understand the potential receptors on HCEC membrane that bind the peptides of interest. Pulling-down of HCEC membrane proteins using peptide Pc-C as anchor followed by LC-MS/MS analysis of the resulting protein preparations revealed seven molecules as promising candidates in this model (Table 2).

**Table 2:** Membrane proteins that potentially bind peptide Pc-C.

Accession name and identification	Mascot Score	GenBank Definition
2NVU_B	129	Chain B, Structure Of Appbp1-Uba3∼nedd8-Nedd8-Mgatp-Ubc12(C111a), A Trapped Ubiquitin-Like Protein Activation Complex
gi|126031226		
AAA86640	78	small GTP binding protein Rab7
gi|1174149		
CAA25855	72	alpha-tubulin
gi|37492		
AAH08938	64	Histocompatibility (minor) 13
gi|14286280		
NP_004037	57	ATP synthase subunit alpha, mitochondrial precursor
gi|4757810		
XP_933678	43	PREDICTED: POTE ankyrin domain family member I isoform 2
gi|88953571		
CAA45026	43	mutant beta-actin (beta'-actin)
gi|28336		

## Discussion

This study used the PhD library screening technique to identify peptides that bind to HCEC surfaces, and confirmed their ability to inhibit *A.f* conidia adherence to corneal epithelial cells in different models. To the best of the authors' knowledge, this is only the second report to use PhD strategies in ocular infection studies. Tiwari *et al* performed PhD screening against heparan sulfate, a molecule that mediates herpes virus infection [26], and used the resulting peptides to effectively manage experimental herpes virus infection of the corneas [27]. The current study used whole corneal epithelial cells as starting targets, providing a more flexible and informative platform for building a panorama of the host-pathogen interactions than those studies using simple target molecules such as extracelluar matrix components [28], host cell surface receptors [29], or protein complex [30].

The significance of the current study is related to the following aspects. First, it directly showed that the PhD-selected peptides could be used as a supportive therapy for managing corneal infections by blocking the binding sites on corneal epithelial cells that otherwise would be bound by pathogens. Due to the complexity of ligand-receptor pairs involved in host-pathogen interactions, the affinities of peptides for HCEC are not necessarily always in good proportion to their efficacies to inhibit pathogen binding to HCEC. This might help to explain the observation that Pc-C and Pc-E had similar inhibitory effects on *A.f* binding to HCEC (Fig. 3) but displayed different inhibitory efficacies on *A.f* adhesion to cornea in both *ex vivo* and *in vivo* models (Fig. 5). If more than one ligand-receptor pair should be involved in the host-pathogen crosstalk, the relative contribution of each ligand-receptor pair to the total host-pathogen adhesion force should be different from each other. Other factors, such as the species difference of HCEC (human) and animal corneas (murine), or certain constituents like extracellular matrix proteins that are present in whole corneas but absent in HCEC cultures, may also contribute to the differential effects of peptides on HCEC and *in vivo* models.

Besides blocking host-pathogen adhesion, the ability of the PhD peptides to stimulate host cell cytokines and fungicidal factors production might also be beneficial for anti-fungal response although this study did not pursue along this direction. IL-6 or IL-8 might be involved in the directed killing of *A.f*, but other factors should play dominant roles in such activity since the fungicidal activity detected in culture medium did not correlate with IL-6 or IL-8 production (Fig. 2). Recent years have seen much progress on dissecting host-pathogen interactions through studies on receptors that recognize various pathogens associated molecular patterns (PAMP), such as Toll-like receptors (TLR). Since MyD88 is critical for the generation of infectious keratitis via mediating TLR signaling [31]–[34], increased expression of MyD88 in peptides-treated HCEC suggests that TLRs pathways might also be involved in the response to these PhD peptides. Quick and abundant upregulation of IL-17 expression in HCEC upon peptide treatment was in line with the previous report that various stress stimuli induced IL-17 induction by HCEC [35]. In the light of IL-17's pathogenic roles in keratitis [36], [37], the contribution of peptide-induced cellular responses to the overall pathogenesis of FK deserves further investigation.

When interpreting the results of peptides binding to cells and designing potential applications for the peptides, the relative binding specificity of the peptides for various cell types should be considered. Though the peptides were obtained by selecting against HCEC, some of them also bind HTK and HUVEC cells to various extents (Fig. 1B), suggesting that the affinity of each peptide depends on the target cell type. Thus, the potential usefulness of these peptides in treating infections in other tissues (like intestine or bronchial epithelium) should be investigated. The high affinity of Pc-B and Pc-E for HTK suggests that they might be used for preventing pathogen binding to corneal stromal cells as well. Similarly, although the current infection models used only *A.f*, the possible efficacy of these peptides for preventing infections of other pathogens could not be excluded. For example, besides being homologue to Alb1p, Pc-B (VATPVPPTLTPF) is highly homologue to Flagellin E (YP_001349650, homologue site at aa168–174 PPTVTPF, score 22.3) of *Pseudomonas (P.) aeruginosa*. Pc-F was also homologue to Flagellin E at aa303–309 (TPPTYAW, homologue score 25.7). Similarly, Pc-D and Pc-I were homologue to different parts of a putative branched-chain amino acid transport protein AzlC of *P. aeruginosa* (YP_002440872) at aa15–21 (APMTAHD, score 20.2) and aa218–226 (SHWQWSSAL, score 23.1) respectively. These results implied that the obtained peptide sequences might also have biological significance in *P. aeruginosa* or other herein unmentioned pathogens. Studies in this direction will not only reveal whether such peptides could be utilized for interfering with *P. aeruginosa* adhesion to HCEC, but also provide clues for studying the functions of putative proteins such as PA15_29901 and AzlC. Surely, caution has to be taken when applying the current observations to any pathogens or any host cells, since it has been clearly shown that the response of hosts depends on the type of pathogens, encountering cells, or even the routes for them to encounter each other [38].

Lastly, the primary result of the pulling-down assay with Pc-C demonstrated the potential power of PhD selected peptides for identifying host receptors. Though none of the revealed binding proteins (Table 2) belongs to the traditional pathogen-binding cellular receptors like TLRs, sequence analysis showed that some if not all of them are potential partners for binding exogenous ligands (peptides in this case). For example, among the total 805 amino acid (aa) length of 2NVY_B, the amino section (aa.3∼376) is 99% (367aa/374aa) identical to “Chain A, Crystal Structure Of Human Crfr2 Alpha Extracellular Domain In Complex With Urocortin 3”, a well-recognized receptor mediating stress response [39]. Similarly, recent studies demonstrated that Rab7 in shrimp functions as the receptor for certain virus [40], and alpha-tubulin binds peptidoglycan during bacterial infection [41]. On the pathogen side, the fact that both Pc-A and Pc-B are homologue of two different parts of a same protein (e.g. Alb1p) is very suggestive, and the results that Pc-A and Pc-B blocked binding of wild-type but not Alb1p-deficient *A. fumigaturs* to HCEC or corneas are confirmative. Though localization of Alb1p in cells is not documented elsewhere, the current study and its involvement in cell wall formation [24], [25] imply that it might be localized in the cellular membrane or cell wall and is directly involved in host-pathogen interactions, but this need verification with other more intensive methodology.

In summary, this study provided evidence to support that PhD could be utilized for studying host-pathogen interactions, especially at the adhesion stage, and for developing prophylactic agents for infectious diseases. While the main body of knowledge about host responses to pathogens has been obtained by looking at well-identified individual receptors, PhD-selection against intact cells greatly increases the chance to identify novel receptors on hosts, novel ligands on pathogens, and novel pathways among them. As such, progress toward further understanding host-pathogen interactions and toward developing therapeutics for infectious diseases will be accelerated.

## Materials and Methods

### Ethic statement

All animal experiments were carried out in accordance with The Chinese Ministry of Science and Technology Guidelines on the Humane Treatment of Laboratory Animals (vGKFCZ-2006-398) and the Association for Research in Vision and Ophthalmology (ARVO) Statement for the Use of Animals in Ophthalmic and Vision Research. This study and all protocols concerning animals were approved by the Shandong Eye Institute Review Board with a permit number SEIRB-2009-2009CB526506.

### Cells, fungal strains, and culture conditions

SV40-immortalized human corneal epithelial cell (HCEC) line (ATCC CRL-11135) was cultured in Dulbecco's modified Eagle's medium (DMEM)/F12 medium with 10% heat-inactivated fetal bovine serum (FBS) (Invitrogen, Carlsbad, CA, USA) at 37°C, in a humidified atmosphere of 5% CO_2_/95% air. The telomerase-immortalized human corneal stromal fibroblasts (HTK cell line), a kind gift of Dr. Jester [42], and human umbilical vascular endothelial cells (HUVEC) were maintained in DMEM supplemented with 10% FBS. A standardized wild type *A.f* strain CGMCC 3.772, purchased from China General Microbiologic Culture Collection Center (Beijing, China), was used in all experiment if not specified else wise. *A.f* strains B-5233 (a clinical isolate) and B-5233/RGD12-8 (an *alb1* disruptant with deficient of nucleotide 2503 to 4070 in polyketide synthase *alb1 g*ene) were gift of Dr. Kwon-Chung [25]. For preparing conidia, all *A.f* strains were cultured on Sabouraud's agar (Haibo, Qingdao, China) at 28°C for 5 days, and the fungal conidia were harvested into sterile saline solutions, which were then adjusted to the proper concentrations.

### Panning of the phage-displayed peptide library against HCEC

Ph.D.-12™ phage display library (New England Biolabs, Beverly, MA, USA) containing 12-mer random peptides fused to the amino terminus of the minor envelope protein pIII was panned against cultured HCEC layers according to previously published protocols [43], [44]. Briefly, HCEC were grown to 80–90% confluence in 60 mm cell culture dishes. After starving in serum-free DMEM/F12 for 2 hours at 37°C, the cells were incubated for 1 hour with 2 mL of phage mixture containing 4×10^10^ PFU phages in 0.5% BSA-PBS. After removal of the phage solution, the cells were rinsed six times with 2 mL 0.5% BSA-PBS supplemented with 0.1% Tween-20 (PBST), followed by a 10 min elution with 1 mL of 0.1 M glycine–HCl (pH 2.2). The cells lysate, now containing bound phages and referred to as sub-library, were harvested into Eppendorf tubes and neutralized with 150 µL 1 M Tris–Cl (pH 9.1). The sub-library was incubated with the *Escherichia coli* strain ER2738 from the Ph.D. -12™ PhD library kit for amplification and titration according to the kit protocol. The amplified sub-library was subjected to 2 more rounds of panning. After the third round of panning, any nonspecific binding phages, namely those that bind either plastic surfaces or the blocking solution components, were removed by culturing the recovered phage mixture for 1 hour at 37°C in a plate that was pre-blocked with 0.5% BSA but contained no cells. The isolation of the specific phages in the supernatant was carried out during the following titration.

### Phage DNA sequencing, bioinformatics analysis, and synthesize of peptides

The selected phages were precipitated with PEG/NaCl after amplification, and single-stranded phage DNA was prepared for sequencing. Briefly, the phage precipitation was lysed with iodide buffer (4 M NaI, l0 mM Tris-HCl, 1 mM EDTA, pH 8.0) and the DNA was precipitated with ethanol, washed with ice cold 70% ethanol, and then re-suspended in TE (l0 mM Tris-HCl. 1 mM EDTA, pH 8.0). DNA sequencing for the displayed peptide was performed by GenScript Nanjing Co., Ltd. (Nanjing, Jiangsu, China) using the NEB Ph.D.-12™ -96 primer (5′-CCCTCATTAGCGTAACG-3′). The resulting DNA sequence was translated into an amino-acid sequence and the corresponding 12-mer peptide sequence was analyzed by the online NCBI BLASTp tool. The retrieved homologue sequences in *Aspergillus fumigatus* were then annotated using the DAVID program [45]. The gene categories or pathways with an expression analysis systematic explorer (EASE) [46], [47] score of 0.05 or less were considered enriched in the genes corresponding to the homologue sequences.

Peptide sequences that showed high homology with biologically relevant pathogen proteins (either characterized or putative) were chemically synthesized (Bootech Bioscience and Technology Co., Ltd, Shanghai, China). The N-terminus of each peptide was modified with biotin and C-terminus with NH_2_ residue. All peptide preparations were over 98% in purity as confirmed by analytical HPLC and electrospray mass spectrometry (data not shown), and were readily soluble in an aqueous medium. In some experiments, a control peptide designed according to the bovine serum albumin sequences, namely pBSA (DMADCCEKQEPE) was used for comparison.

### ELISA assay of phages or peptides binding to cells

After reaching 80–90% confluence in a 96-well plate, cells were starved in serum free DMEM/F12 for 2 hours, and then incubated with 0.5% BSA in PBST for 1 hour, followed by 6 washes with PBST. Pure 10^12^ PFU amplified phages or 100 µM synthesized peptides were added to 3 wells of each cell, and incubated at 37°C for an additional 1 hour. Unbound phages or peptides were removed by 6 washes with PBST. Then horse radish peroxidase (HRP)-conjugated anti-M13 antibodies were added to the phage incubation groups, or HRP-conjugated streptavidin (BD Biosciences, San Jose, CA, USA) was added to peptide incubation groups, for another 1 hour, followed by 6 washes. Tetramethyl benzidine (BD Biosciences, San Jose, CA, USA) was added at room temperature in the dark for 30 minutes, and 1N H_3_PO_4_ was added to stop the reaction. The absorbance was read at 405 nm using spectramax M2 microplate reader (Molecular Devices, Sunnyvale, CA, USA). Each treatment was tested in triplicate.

### Effect of synthetic peptides on HCEC survival and cytokine production

To monitor the potential toxicity of peptides to HCEC, the cells were incubated in the presence of peptides for 72 hours, followed by a 4 hour incubation with 3-(4, 5)-dimethylthiahiazo (-z-y1)-3,5-di-phenytetrazoliumromide (MTT). Benzalkonium Bromide 0.01% was used as a positive toxic control. The MTT-transformed crystals were dissolved in dimethyl sulfoxide, and the absorbance at 490 nm was measured using a microplate reader. Each treatment was repeated for three times.

To measure the effect of peptides on the expression of genes of interest, HCEC were stimulated with peptides for 1 hour or 4 hours in triplicates. Total RNA was extracted from cells using NucleoSpin® RNA II Kit (MACHEREY-NAGEL, Düren, Germany) and reverse transcribed into first strand cDNA using a PrimeScript™ 1st strand cDNA Synthesis Kit (Takara, Otsu, Japan). Quantitative real-time PCR was performed using Taqman reagents and the Applied Biosystems 7500 Real-Time PCR System (Applied Biosystems, Foster City, USA) according to the instructions of the manufacturer. The specific primers and probes of cytokines used in this study are listed in Table 3. Cycling conditions were 10 s at 95°C, followed by 40 two-step cycles (15 s at 95°C and 1 min at 60°C). Data were analyzed with the SDS System Software (Applied Biosystems) using hB2-M as reference gene.

**Table 3:** Sequences of primers and probes for real time-PCR.

Genes (accession number)	Primer and probe sequence
hB2-M	F, 5′-TAGCTGTGCTCGCGCTACTCT-3′
(NM_004048)	R, 5′-TTCTCTGCTGGATGACGTGAGTAA-3′
	Probe, 5′-CTGGAGGCTATCCAGCGTACTCCA-3′
hMyD88	F, 5′-GCTATTGCCCCAGCGACAT-3′
(NM_002468)	R, 5′-CGGTCAGACACACACAACTTCA-3′
	Probe, 5′-CAGTTTGTGCAGGAGATGATCCG-3′
hIL-8	F, 5′-GGCAGCCTTCCTGATTTCTG-3′
(NM_000584)	R, 5′-TGCACTGACATCTAAGTTCTTTAGCA-3′
	Probe, 5′-TGTGTGAAGGTGCAGTTTTGCCAAGG-3′
hIL-6	F, 5′-CCCCCAGGAGAAGATTCCAA-3′
(NM_000600)	R, 5′-TCAATTCGTTCTGAAGAGGTGAGT-3′
	Probe, 5′-ATGTAGCCGCCCCACACAGACAG-3′
hIL-17	F, 5′-GCCATAGTGAAGGCAGGAAT-3′
(NM_002190)	R, 5′-CAGGTTGACCATCACAGTCC-3′
	Probe, 5′-TCCCACGAAATCCAGGATGCC-3′

Note: Probes were labeled with FAM and TAMRA at 5′ and 3′ end respectively.

### Effect of HCEC culture supernatant on survival of pathogen in vitro

The supernatants of HCEC, stimulated with each peptide for 1 hour, were collected from each well and tested for their effect on the survival of *A.f.* In brief, *A.f* conidia were adjusted to 5×10^7^ CFU/mL and seeded into 96-well plates at 10 µL/well, and the supernatants from different peptide stimulated HCEC were added at 100 µL/well. Culture medium containing the starting concentration of peptides was also used as control to check whether peptides per se have any effect on fungal growth or survival. Triplicate wells were set for each supernatant or medium sample. After culture at 33°C with shaking (150 rpm) for 2 hours, 10 µL of propidium iodide (PI, 50 µg/mL) was added to each well for 10 minutes to stain the dead cells. The plates were spun at 1000 rpm for 5 minutes and the cells were viewed by using a Nikon confocal laser scanning microscope. The numbers of killed conidia and total conidia in five randomly selected fields were counted and the fungicidal rates of the culture supernatant were calculated.

### Effects of peptides on *Aspergillus* adherence to HCEC

When HCEC grown in 24-well plates formed a confluent monolayer at 37°C, the medium was changed to serum free DMEM/F12 for 2 hours, and then 500 µL of 100 µM peptides were added for 1 hour of incubation, with normal saline as negative control. Then 10^7^ CFU *A.f* conidia were added to each well for 1 hour at 37°C, and the cells were washed 3 times with normal saline to removed unbound conidia. One milliliter lysis buffer (0.25% trypsin, 0.02% EDTA, 0.01% Triton X-100) was added to each well for 10 minutes, and the samples were diluted by 10-folds, spread on Sabouraud's agar plates, and cultured at 37°C for 48 hours. The resulting colonies were counted. Three duplications were set in each group. For some peptides, inhibition assays using other concentrations (0, 0.01, 0.1, 1, 10 and 100 µM) were also performed and a dose-response plot was obtained, from which the 50% inhibitory concentration (IC50) was calculated.

### Effect of peptides on *Aspergillus* adherence to cornea ex vivo

Inhibition assays at the eye organ model level were performed as described previously [48]. In brief, Balb/C mice were killed after anesthesia, their corneal epithelia were scarified with a 26-G syringe needle for “+” to mimic the situation of wounds occurred to the corneas. The eyes were enucleated, washed with a serum-free saline buffer, and placed on 5% agar in 96-well plates with the corneas facing up. Peptides selected from above assays were added to each well to 100 µL at 100 µM, with normal saline and Natamycin Eye Drops (NATACYN®, 5% Natamycin in 0.02% benzalkonium chloride, pH 7.0) as negative and positive controls respectively. Three eyes were included in each group. After 1 hour incubation, 10^7^ CFU *A.f* conidia were added to each well the incubation continued for another 1 hour, all at 37°C. The eyeballs were then washed three times with normal saline, and the corneas were excised along the limbal line. The corneas were placed in 0.5 mL saline and homogenated by ultrasonication. The pathogen loads in the samples were determined as described above.

### Effect of peptides on Aspergillus adherence to cornea in vivo

Balb/C mice, 6∼8 weeks old, were anesthetized and their corneas were blotted with filter paper as described [49] to achieve maximal adhesion bed for the pathogens. In brief, a piece of filter paper was used to gently wipe over the corneal surface. With practice, this method ensured removal of the squamous layer of the epithelium as confirmed by histology (data not shown). A plastic tube of 3 mm in inner diameter and 1 cm in length was sleeved around the eyeball and fixed by sutures in the eyelid. Ten microliters of infection mixture containing 10^7^ CFU of *A.f* and 100 µM peptide were added into the tube for 1 hour to allow infection. The mice were euthanized, and the eyes were enucleated and washed three times with normal saline. The fungi adhered to the corneas were quantified using CFU assay as described above. Again, saline buffer and Natamycin Eye Drops were used as controls. Four mice were included in each group, and the assay was performed 3 times.

### Effect of peptides on keratitis caused by *Aspergillus*

The potential effect of peptides on FK development were determined using a similar model described previously [50] with modifications. In brief, the corneas of Balb/c mice were scarified as above and the wounded corneas received a 5 µL drop of 100 µM peptide 4 times during 1 hour before infection. Corneas received normal saline and Natamycin Eye Drops as controls. The eyes were topically inoculated with 10 µL (10^7^ CFU) *A.f* conidia using the same method described in the *in vivo* adherence model. After 1 hour of infection, the application of peptides or controls continued hourly for 5 hours. Fifty mice were included per treatment group. On days 1, 3, 7, 10, and 14, post infection, the diseases of the corneas were examined with a slit lamp microscope. The scoring system used was essentially that described by Wu [51]. The load of *A.f* in the corneas on days 1, 3, 5, 7, and 10 was tested as described in the *ex vivo* model section. For histopathology assay, the enucleated eyeballs were fixed in neutral phosphate-buffered formalin (10%) for at least 24 hours, followed by routine histology procedure for Hematoxylin-Eosin (HE) staining and light microscopic evaluation.

### In vitro antifungal activity of Natamycin in the presence of peptides

The potential use of peptides in combination with Natamycin was determined by measuring the minimum inhibitory concentration (MIC) of Natamycin in the in vitro coculture model following the merit of Clinical and Laboratory Standards Institute (CLSI) M38-A document with modification. After incubation of the monolayer HCEC in serum free DMEM/F12 for 2 hours in 96-well plates, 100 µL of peptides with concentration of 0, 0.01, 0.1, 1, 10, 100 µM were respectively added to each row of the plate for 1 hour incubation. Then 10^6^ CFU *A.f* conidia were added to each well for another 1 hour at 37°C, followed by 3 washes with normal saline to remove the unbound conidia. Natamycin with different concentrations (64, 32, 16, 8, 4, 2, 1, 0.5, 0.25, 0.125, 0.0625, 0 µg/mL) was added to each column of the plate. The plates were cultured at 37°C for 48 hours and examined with nude eyes for the presence or absence of fungal growth. The lowest concentration of Natamycin that gave no fungal growth was recorded as the MIC.

### Pull-down assay of HCEC membrane proteins with peptide Pc-C

About 10^8^ HCEC cells were collected for preparation of membrane proteins using a Membrane Protein Extraction Kit (Bestbio, Shanghai, China) following the protocol from the manufacture. As measured by a BCA protein assay kit (Beyotime, Shanghai, China), the total quantity was about 1.5 mg. To prepare the affinity matrix, 500 µL of 100 µM peptide Pc-C with N-terminal biotin modification were incubated with 100 µL of streptavidin-mobilized agarose CL-4B (sigma, St. Louis, MO, USA) at 4°C for 6 hours on a rotator. Unbound Pc-C peptides were removed by four washes with PBS. Subsequently, the extracted membrane proteins in 300 µL of PBS were incubated with the agarose at 4°C for 8 hours with rotation. Following five washes with PBS, the proteins bound to the agarose were eluted by 100 µL of 0.2 M Glycin-HCl. After immediate neutralization with 15 µL of 1 M Tris-Cl, pH 9.1, the elution was condensed to 20 µL using ultrafiltration tube (Millpore, Bedford, MA, USA) and resolved on a 12% SDS-PAGE separation gel for 30 minutes. The gel was stained with G-250 dye, destained with 0.1% acetic acid, and cut into 4 pieces according to their molecular weight (Figure S1). After in-gel digestion with trypsin, the samples were subjected to routine liquid chromatography-tandem mass spectrometry (LC-MS/MS) on Synapt™. G2 HDMS (Waters corporation, Milford, MA, USA). The processed spectra were run against protein NCBInr homo sapiens databases using Mascot search engine (Matrix Science Inc., Boston, MA, USA). A score over 39 suggested a match between the input mass spectrum with a database sequence [39].

### Statistical analysis

The data are presented as means±SD. SPSS (SPSS software, 11.5 version) was used for data processing. The statistical significance of cytotoxity, the inhibitory effect on *A.f* adherence to HCEC, and corneal epithelia, fungal load, and clinical scores in the mice FK model was determined with a Multiple Comparison, one-way analysis of variance (ANOVA). *P*<0.05 was considered statistically significant.

## Supporting Information

Figure S1 **SDS-PAGE of membrane proteins pulled-down by peptide Pc-C.** Extracted membrane proteins were incubated with Pc-C binding agarose and the proteins bound by Pc-C were eluted, neutralized, condensed and subjected to 12% SDS-PAGE gels. After separation, the proteins in the gel were identified by LC-MS/MS.
(DOC)

Table S1 **Peptide and DNA sequences corresponding to the phages that bind HCEC and their homology with A. fumigatus proteins.**
(DOC)

Table S2 **Summary of defined/hypothetical proteins that are homologue to Pc-A peptide (ATKVKIPFEAKV) with high scores.** Detailed homology analysis results obtained with other peptides (namely Pc-B∼N) are not shown here either, since it will be more convenient for the readers to do blastp analysis for themselves.
(DOC)

Table S3 **Enriched gene functions and pathways all proteins of Aspergillus fumigatus that show high homology with 14 peptides.** The first 100 homologue sequences that gave the highest homologue scores for each of the 14 peptides were combined and subjected to DAVID analysis.
(DOC)
