# Supplementary material for: Phage Display against Corneal Epithelial Cells Produced Bioactive Peptides That Inhibit Aspergillus Adhesion to the Corneas
Source: PLoS One. 2012 Mar 12;7(3):e33578. doi: 10.1371/journal.pone.0033578 (PMC3299800; doi:10.1371/journal.pone.0033578)
Supplement: Figure S1 — SDS-PAGE of membrane proteins pulled-down by peptide Pc-C. Extracted membrane proteins were incubated with Pc-C binding agarose and the proteins bound by Pc-C were eluted, neutralized, condensed and subjected to 12% SDS-PAGE gels. After separation, the proteins in the gel were identified by LC-MS/MS. (DOC) [file pone.0033578.s001.doc]

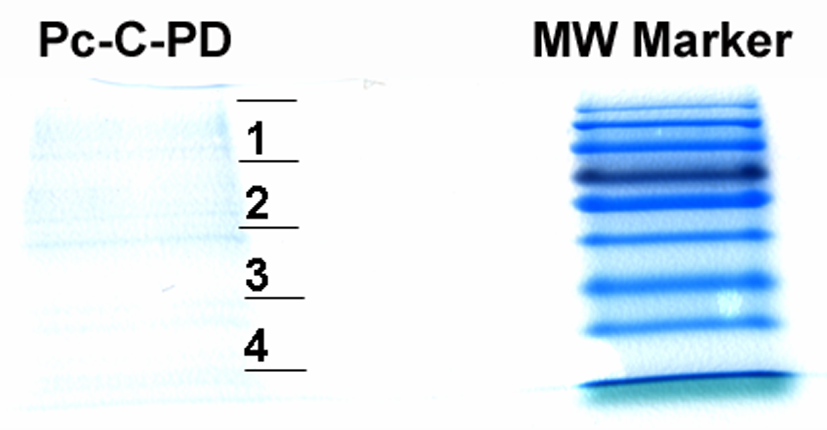


**Figure S1. SDS-PAGE of membrane proteins pulled-down by peptide Pc-C.**

About 1 mg extracted membrane proteins were incubated with 100 μL of Pc-C binding agarose at 4 °C for 8 hours of rotation. After 5 times of wash with PBS, the proteins specific binding to Pc-C were eluted by 100 μL of 0.2M Glycin-HCl, and immediately neutralized by 15 μL of 1M Tris-Cl (pH=9.1). The elution was condensed to 20 μL and loaded onto 12% SDS-PAGE gels. After separation, the gel containing protein sample was divided into 4 pieces and identified by LC-MS/MS.
