# Supplementary material for: Phage Display against Corneal Epithelial Cells Produced Bioactive Peptides That Inhibit Aspergillus Adhesion to the Corneas
Source: PLoS One. 2012 Mar 12;7(3):e33578. doi: 10.1371/journal.pone.0033578 (PMC3299800; doi:10.1371/journal.pone.0033578)
Supplement: Table S1 — Peptide and DNA sequences corresponding to the phages that bind HCEC and their homology with A. fumigatus proteins. (DOC) [file pone.0033578.s002.doc]

Table S1. Peptide sequences that bind HCEC and show homologue to *A. fumigatus* proteins

| Peptide code and sequence | *Aspergillus* fumigatus | |
| --- | --- | --- |
|  | homologue sequence description | BLAST alignment |
| Pc-A  ATKVKIPFEAKV (CACCTTCGCCTCAAAAGGAATCTTCACCTTAGTAGC) | ACJ13039  polyketide synthase Alb1p | TKVKIPF 8  TK K+PF  TKLKVPF 1097 |
|  | EDP49269  NACHT and Ankyrin domain protein | TKVKIP 7  TK+KIP  TKIKIP 1089 |
|  | EDP47502  HATPase_c domain protein, putative | ATKVKIPFE 9  ATKV I FE  ATKVTIKFE 66 |
|  | EDP50664  glutamine synthetase | VKIPFEAK 11  VKI FE K  VKISFEPK 226 |
| Pc-B  VATPVPPTLTPF  (AATCGGAGTCAGAGTCGGCGGAACCGGCGTCGCAAC) | ACJ13039  polyketide synthase Alb1p | VATPVPPT 8  V TPVP T  VTTPVPAT 902 |
|  | XP_749784  hypothetical protein AFUA_1G00320 | VATPVPPTLT 10  VATP PPTLT  VATP-PPTLT 74 |
|  | EDP47447  sodium transporting ATPase, putative | TPVPPT 8  TPVPPT  TPVPPT 73 |
| Pc-C  ATLRTYPYMDRA  (AGCCCGATCCATATAAGGATACGTACGCAGCGTAGC) | XP_749787  cell surface metalloreductase, putative | RTYPY 8  RTYPY  RTYPY 105 |
|  | XP_750165  aspartate aminotransferase, putative | RTYPY 8  RTYPY  RTYPY 187 |
|  | EDP54828  bifunctional tryptophan synthase TRPB | YPYMDR 11  YPYM R  YPYMGR 387 |
| Pc-D  QLAPMATHDKHP  (CGGATGCTTATCATGAGTAGCCATCGGAGCAAGCTG) | XP_746369  antigenic cell wall galactomannoprotein, putative | QLAPM 5  QLAPM  QLAPM 167 |
|  | XP_751254  MFS multidrug transporter | M-ATHDKHP 12  M A HD HP  MSASHDSHP 9 |
| Pc-E  YALRPGMPQWLE  (AGCACGCGTAGGATTAGGAAACGGCGACTCCGCATG) | XP_750173  endo-1,3-beta-glucanase Engl1 | M-PQWLE 12  M PQWLE  MDPQWLE 738 |
|  | EDP50333  ZIP Zinc transporter, putative | ESPFPNPT 10  E PFP PT  ETPFPTPT 50 |
|  | EDP53374  MFS multidrug transporter, putative | SPFPN 8  SPFPN  SPFPN 88 |
|  | XP_747279  oligopeptidase family protein | PQWLE 12  PQWLE  PQWLE 75 |
| Pc-F  TPPTYSWFTHRM  (CTCAAGCCACTGCGGCATACCAGGCCTCAACGCATA) | XP_751660  Leucine rich repeat domain protein | PTYSWFT 9  PTYS FT  PTYSRFT 20 |
|  | XP_746913  Putative polyketide synthase | PTYSW 7  PTYSW  PTYSW 1273 |
|  | XP_754859  exo-beta-1,3-glucanase, putative | PPTYS 6  PPTYS  PPTYS 440 |
| Pc-G  GSATNPTMGQRM  (CGCAATAGACGAATTCGAATGCAAAGTAATCTTATT) | EDP54833  hypothetical protein AFUB_028930 | SATNPT 7  SATNPT  SATNPT 315 |
|  | XP_001481671  GYF domain protein | SATNPT 7  SATNPT  SATNPT 315 |
|  | XP_747950  alpha-1,3-glucanase | GSA-----TNPTMGQ 10  GSA NP MGQ  GSADGTTGNNPAMGQ 350 |
| Pc-H  AETHVLNKHTPL  (ACGCCGCCTACGTATTCTTGGTTTACTCATCGTATG) | XP_752425  GPI anchored protein, putative | HVLNKHTP 11  H LNKH P  HALNKHAP 85 |
|  | XP_747678  GAS2 domain protein | HVLNKHTP 11  HVLN H P  HVLNAHIP 181 |
| Pc-I  HSSSHWSWSTPL  (GGTTCGGCTACTAATCCGACGATGGGTCAGCGGATG) | XP_753005  MFS hexose transporter, putative | SSHWSW 8  SS+WSW  SSQWSW 194 |
|  | XP_750940  3-oxoacyl-acyl carrier protein reductase | WSWST 10  WSWST  WSWST 56 |
| Pc-J  NMRLLANPAMAG  (GCTGAGACGCATGTTCTGAATAAGCATACTCCGCTG) | XP_747167  polyketide synthase | NMRLLA 6  NMRLLA  NMRLLA 1967 |
|  | XP_752055  clathrin heavy chain | LLANPAMAG 12  L+ NPAM G  LMENPAMSG 363 |
| Pc-K  QIPAQNRLVFLT  (CGTCAAAAACACCAGACGATTCTGCGCAGGAATCTG) | XP_750702  checkpoint protein kinase (SldA), putative | AQNRLVFLT 12  AQNRL F+T  AQNRLPFMT 662 |
|  | XP_749268  Potassium/sodium P-type ATPase | PAQNRLVF 10  PA NRLVF  PAVNRLVF 1006 |
| Pc-L  VPGWDSHNARHQ  (CTAATGCCGCGCATTATGACTATCCCAACCAGGCAC) | XP_746960  MFS transporter, putative | HNARHQ 12  H ARHQ  HHARHQ 240 |
| Pc-M  HAESPFPNPTRA  (AGCACGCGTAGGATTAGGAAACGGCGACTCCGCATG) | XP_749029  Conserved hypothetical protein | PFPNPT 10  PFPNPT  PFPNPT 507 |
|  | XP_746882  ABC multidrug transporter, putative | AESPF 6  AESPF  AESPF 58 |
| Pc-N  NKITLHSNSSIA  (CGCAATAGACGAATTCGAATGCAAAGTAATCTTATT) | XP_749340  peptidyl-prolyl cis-trans isomerase Cpr7 | TLHSNSS 10  TLHSNSS  TLHSNSS 280 |
|  | XP_755784  conserved hypothetical protein | KITLHSNSSI 11  K LH NSSI  KLQLHANSSI 353 |
