# Supplementary material for: Phage Display against Corneal Epithelial Cells Produced Bioactive Peptides That Inhibit Aspergillus Adhesion to the Corneas
Source: PLoS One. 2012 Mar 12;7(3):e33578. doi: 10.1371/journal.pone.0033578 (PMC3299800; doi:10.1371/journal.pone.0033578)
Supplement: Table S2 — Summary of defined/hypothetical proteins that are homologue to Pc-A peptide (ATKVKIPFEAKV) with high scores. Detailed homology analysis results obtained with other peptides (namely Pc-B∼N) are not shown here either, since it will be more convenient for the readers to do blastp analysis for themselves. (DOC) [file pone.0033578.s003.doc]

**Table S2. Information for defined/hypothetical proteins that are homologue to Pc-A peptide (ATKVKIPFEAKV) with high scores***

| **Protein**  **Accession** | **Description，Genbank identification number and [species]** | **Max score** | **E value** | **Alignment** |
| --- | --- | --- | --- | --- |
| ZP_03677039.1 | hypothetical protein BACCELL_01375 [Bacteroides cellulosilyticus DSM 14838]  >gb|EEF90962.1| | 28.6 | 8.7 | Query 1 ATKVKIPFEAK 11  ATKVKI F AK  Sbjct 682 ATKVKIAFNAK 692 |
| EGR30881.1 | hypothetical protein IMG5_121790 [Ichthyophthirius multifiliis] | 28.6 | 8.8 | Query 3 KVKIPFEAK 11  KVKIPFE K  Sbjct 84 KVKIPFETK 92 |
| XP_002786558.1 | signal recognition particle 54 kDa protein 2 [Perkinsus marinus ATCC 50983]  >gb|EER18354.1| | 28.2 | 12 | Query 1 ATKVKIPF 8  ATKVKIPF  Sbjct 153 ATKVKIPF 160 |
| XP_002784438.1 | signal recognition particle 54 kDa protein 2 [Perkinsus marinus ATCC 50983] >gb|EER16234.1| | 28.2 | 12 | Query 1 ATKVKIPF 8  ATKVKIPF  Sbjct 153 ATKVKIPF 160 |
| XP_628344.1 | SRP54. signal recognition 54. GTpase. [Cryptosporidium parvum Iowa II]  >gb|EAK90603.1| | 28.2 | 12 | Query 1 ATKVKIPF 8  ATKVKIPF  Sbjct 167 ATKVKIPF 174 |
| XP_002964268.1 | hypothetical protein SELMODRAFT_82182 [Selaginella moellendorffii]  >gb|EFJ34601.1| | 28.2 | 12 | Query 1 ATKVKIPF 8  ATKVKIPF  Sbjct 150 ATKVKIPF 157 |
| XP_002989541.1 | hypothetical protein SELMODRAFT_129975 [Selaginella moellendorffii]  >gb|EFJ09417.1| | 28.2 | 12 | Query 1 ATKVKIPF 8  ATKVKIPF  Sbjct 150 ATKVKIPF 157 |
| XP_002964266.1 | hypothetical protein SELMODRAFT_81201 [Selaginella moellendorffii]  >gb|EFJ34599.1| | 28.2 | 12 | Query 1 ATKVKIPF 8  ATKVKIPF  Sbjct 150 ATKVKIPF 157 |
| AEM21840.1 | hypothetical protein Bint_1219 [Brachyspira intermedia PWS/A] | 28.2 | 13 | Query 3 KVKIPFEAKV 12  K+KI FEAKV  Sbjct 21 KIKISFEAKV 30 |
| XP_001197769.1 | PREDICTED: similar to twitchin, partial [Strongylocentrotus purpuratus]  >ref|XP_793336.2| | 28.2 | 13 | Query 3 KVKIPFEAK 11  KVKIPFE K  Sbjct 20 KVKIPFEGK 28 |
| AAD51793.1 | Gag-Pro-Pol-Env protein [Homo sapiens] | 27.4 | 25 | Query 4 VKIPFEAKV 12  +KIPFEAK+  Sbjct 916 IKIPFEAKI 924 |
| AAD51791.1 | Gag-Pro-Pol protein [Homo sapiens] | 27.4 | 25 | Query 4 VKIPFEAKV 12  +KIPFEAK+  Sbjct 916 IKIPFEAKI 924 |
| P63128.3 | Gag-Pro-Pol protein [Homo sapiens], Reverse transcriptase/ribonuclease H;  >gb|AAD51799.1|AF164615_1 | 27.4 | 25 | Query 4 VKIPFEAKV 12  +KIPFEAK+  Sbjct 916 IKIPFEAKI 924 |
| ZP_06807051.1 | sensor histidine kinase [Aerococcus viridans ATCC 11563]  >gb|EFG50564.1| | 27.4 | 25 | Query 2 TKVKIPFEAKV 12  TKVKI FEA V  Sbjct 235 TKVKINFEATV 245 |
| CAA71419.1 | protease [Human endogenous retrovirus K] | 27.4 | 25 | Query 4 VKIPFEAKV 12  +KIPFEAK+  Sbjct 315 IKIPFEAKI 323 |
| ABA28251.1 | protease [Human endogenous retrovirus K] | 27.4 | 25 | Query 4 VKIPFEAKV 12  +KIPFEAK+  Sbjct 194 IKIPFEAKI 202 |
| ABA28254.1 | protease [Human endogenous retrovirus K] | 27.4 | 25 | Query 4 VKIPFEAKV 12  +KIPFEAK+  Sbjct 194 IKIPFEAKI 202 |
| ABA28243.1 | protease [Human endogenous retrovirus K]  >gb|ABA28244.1, ABA28245.1| | 27.4 | 25 | Query 4 VKIPFEAKV 12  +KIPFEAK+  Sbjct 194 IKIPFEAKI 202 |
| ABA28246.1 | protease [Human endogenous retrovirus K] | 27.4 | 25 | Query 4 VKIPFEAKV 12  +KIPFEAK+  Sbjct 194 IKIPFEAKI 202 |
| P63127.1 | HERV-K_6q14.1 provirus ancestral Pro protein | 27.4 | 25 | Query 4 VKIPFEAKV 12  +KIPFEAK+  Sbjct 137 IKIPFEAKI 145 |
| P63122.1 | HERV-K_8p23.1 provirus ancestral Pro protein | 27.4 | 25 | Query 4 VKIPFEAKV 12  +KIPFEAK+  Sbjct 137 IKIPFEAKI 145 |
| P63129.1 | HERV-K_22q11.21 provirus ancestral Pro protein | 27.4 | 25 | Query 4 VKIPFEAKV 12  +KIPFEAK+  Sbjct 137 IKIPFEAKI 145 |
| ZP_08332549.1 | hypothetical protein HMPREF0992_01473 [Lachnospiraceae bacterium 6_1_63FAA]  >gb|EGG83678.1| | 26.9 | 35 | Query 2 TKVKI--PFEA 10  TKVKI PFEA  Sbjct 702 TKVKILLPFEA 712 |
| YP_002886687.1 | Mannosyl-glycoprotein endo-beta-N-acetylglucosaminidase [Exiguobacterium sp. AT1b]  >gb|ACQ71242.1| | 26.5 | 50 | Query 1 ATKV-KIPFEAKV 12  ATKV IPF AKV  Sbjct 396 ATKVATIPFGAKV 408 |
| ZP_08321456.1 | conserved domain protein [Paraprevotella xylaniphila YIT 11841]  >gb|EGG51879.1| | 26.5 | 50 | Query 2 TKVKIPFEAK 11  TKVKIP EAK  Sbjct 140 TKVKIP-EAK 148 |
| YP_001285797.1 | capsid protein [Phormidium phage Pf-WMP3]  >gb|ABQ12472.1| | 26.5 | 50 | Query 1 ATKVKIPFEAK 11  ATK KIPFE K  Sbjct 45 ATK-KIPFEGK 54 |
| ZP_03476334.1 | hypothetical protein PRABACTJOHN_02002 [Parabacteroides johnsonii DSM 18315]  >gb|EEC96585.1| | 26.5 | 50 | Query 2 TKV----KIPFEAKV 12  TK+ KIPFEA V  Sbjct 76 TKILAALKIPFEANV 90 |
| XP_003082119.1 | unnamed protein product [Ostreococcus tauri]  >emb|CAL55922.1| | 26.1 | 71 | Query 3 KVKIPFE 9  KVKIPFE  Sbjct 667 KVKIPFE 673 |
| XP_003079742.1 | unnamed protein product [Ostreococcus tauri]  >emb|CAL53388.1| | 26.1 | 71 | Query 2 TKVKIPFE 9  T VKIPFE  Sbjct 411 TRVKIPFE 418 |
| XP_972284.1 | PREDICTED: hypothetical protein [Tribolium castaneum] | 26.1 | 71 | Query 2 TKVKIPF-----EAKV 12  TKVKI F EAKV  Sbjct 75 TKVKIEFDEEEIEAKV 90 |
| EFA06690.1 | hypothetical protein TcasGA2_TC009621 [Tribolium castaneum] | 26.1 | 71 | Query 2 TKVKIPF-----EAKV 12  TKVKI F EAKV  Sbjct 75 TKVKIEFDEEEIEAKV 90 |
| YP_001817898.1 | TonB family protein [Opitutus terrae PB90-1]  >gb|ACB74298.1| | 26.1 | 71 | Query 3 KVKIPFE 9  KVKIPFE  Sbjct 452 KVKIPFE 458 |
| ZP_08045493.1 | hypothetical protein ZOD2009_15641 [Haladaptatus paucihalophilus DX253]  >gb|EFW91036.1| | 26.1 | 71 | Query 3 KVKIPFE 9  KVKIPFE  Sbjct 296 KVKIPFE 302 |
| ZP_07526241.1 | efflux transporter, RND family, MFP subunit [Peptostreptococcus stomatis DSM 17678]  >gb|EFM64529.1| | 26.1 | 71 | Query 2 TKVKIPFEAKV 12  TKVK PF+ KV  Sbjct 161 TKVKAPFDGKV 171 |
| XP_001953680.1 | GF17107 [Drosophila ananassae]  >gb|EDV42241.1| | 26.1 | 71 | Query 3 KVKIPFE 9  KVKIPFE  Sbjct 211 KVKIPFE 217 |
| ZP_02065346.1 | hypothetical protein BACOVA_02321 [Bacteroides ovatus ATCC 8483]  >ref|ZP_05415334.1|[Bacteroides finegoldii DSM 17565]  >ref|ZP_08594020.1|[Bacteroides ovatus 3_8_47FAA]  >gb|EDO11827.1|[Bacteroides ovatus ATCC 8483]  >gb|EEX45601.1|[Bacteroides finegoldii DSM 17565]  >gb|EGM96671.1|[Bacteroides ovatus 3_8_47FAA] | 26.1 | 72 | Query 3 KVKIPFEAK 11  KVKI FEAK  Sbjct 142 KVKIEFEAK 150 |
| YP_003071736.1 | phosphoribosylaminoimidazole carboxylase, catalytic subunit [Teredinibacter turnerae T7901]  >gb|ACR13177.1| | 26.1 | 72 | Query 3 KVKIPFEAKV 12  K IPFEAKV  Sbjct 28 KLEIPFEAKV 37 |
| ZP_08709255.1 | G5 domain protein [Peptoniphilus sp. oral taxon 375 str. F0436]  >gb|EGS30898.1| | 25.7 | 101 | Query 5 KIPFEAKV 12  KIPFE KV  Sbjct 813 KIPFETKV 820 |
| ZP_07400404.1 | conserved hypothetical protein [Peptoniphilus duerdenii ATCC BAA-1640]  >gb|EFM24614.1| | 25.7 | 101 | Query 5 KIPFEAKV 12  KIPFE KV  Sbjct 832 KIPFETKV 839 |
| XP_310498.4 | AGAP000576-PA [Anopheles gambiae str. PEST] | 25.7 | 101 | Query 1 ATKVKIPF 8  ATKVK+PF  Sbjct 594 ATKVKVPF 601 |
| EAA06333.5 | AGAP000576-PA [Anopheles gambiae str. PEST] | 25.7 | 101 | Query 1 ATKVKIPF 8  ATKVK+PF  Sbjct 590 ATKVKVPF 597 |
| EFR26892.1 | hypothetical protein AND_06716 [Anopheles darlingi] | 25.7 | 101 | Query 1 ATKVKIPF 8  ATKVK+PF  Sbjct 76 ATKVKVPF 83 |
| ZP_07015651.1 | NADH dehydrogenase (quinone) [Desulfonatronospira thiodismutans ASO3-1]  >gb|EFI35801.1| | 25.7 | 101 | Query 3 KVKIPFEAKV 12  K+KIP+EA V  Sbjct 627 KIKIPYEAMV 636 |
| XP_002996658.1 | hypothetical protein NCER_100221 [Nosema ceranae BRL01]  >gb|EEQ82987.1| | 25.7 | 101 | Query 2 TKVKIPFE 9  T VKIPFE  Sbjct 273 TNVKIPFE 280 |
| YP_004182255.1 | oxidoreductase domain-containing protein [Terriglobus saanensis SP1PR4]  >gb|ADV82261.1| | 25.7 | 101 | Query 2 TKVKIPFEAKV 12  T V +PFEAK+  Sbjct 43 TMVNVPFEAKI 53 |
| YP_290330.1 | putative secretory protein [Thermobifida fusca YX]  >gb|AAZ56307.1| | 25.7 | 101 | Query 4 VKIPFEA 10  VKIPFEA  Sbjct 337 VKIPFEA 343 |
| ZP_07833600.1 | hypothetical protein HMPREF9406_3593 [Clostridium sp. HGF2]  >gb|EFR36820.1| | 25.7 | 101 | Query 1 ATKVK--IPFEAKV 12  A KVK I F+AKV  Sbjct 240 ASKVKQFISFDAKV 253 |
| NP_578133.1 | hypothetical protein PF0404 [Pyrococcus furiosus DSM 3638]  >gb|AAL80528.1| | 25.7 | 101 | Query 4 VKIPFEAKV 12  VK+PFE KV  Sbjct 83 VKVPFEVKV 91 |
| YP_001496302.1 | hypothetical protein A1I_04630 [Rickettsia bellii OSU 85-389]  >gb|ABV79265.1| | 25.7 | 101 | Query 6 IPFEAKV 12  IPFEAKV  Sbjct 74 IPFEAKV 80 |
| YP_537877.1 | hypothetical protein RBE_0707 [Rickettsia bellii RML369-C]  >gb|ABE04788.1| | 25.7 | 101 | Query 6 IPFEAKV 12  IPFEAKV  Sbjct 74 IPFEAKV 80 |
| YP_004628327.1 | chaperone DnaJ domain-containing protein [Thermodesulfobacterium sp. OPB45]  >gb|AEH23399.1| | 25.7 | 101 | Query 5 KIPFEAK 11  KIPFEAK  Sbjct 280 KIPFEAK 286 |
| ZP_08647304.1 | Diaminopimelate epimerase [gamma proteobacterium IMCC2047]  >gb|EGH00275.1| | 25.7 | 101 | Query 5 KIPFEAK 11  KIPFEAK  Sbjct 114 KIPFEAK 120 |
| NP_213799.1 | rare lipoprotein A [Aquifex aeolicus VF5]  >sp|O67235.1, AAC07192.1| | 25.7 | 101 | Query 6 IPFEAKV 12  IPFEAKV  Sbjct 215 IPFEAKV 221 |
| ZP_06161696.1 | 16S rRNA methyltransferase GidB [Actinomyces sp. oral taxon 848 str. F0332]  >gb|EEZ79117.1| | 25.7 | 101 | Query 6 IPFEAKV 12  IPFEAKV  Sbjct 71 IPFEAKV 77 |
| YP_004749944.1 | phosphoribosylaminoimidazole carboxylase catalytic subunit [Acidithiobacillus caldus SM-1]  >gb|AEK59242.1| | 25.7 | 102 | Query 5 KIPFEAKV 12  KIPFEA V  Sbjct 30 KIPFEARV 37 |
| ZP_05292040.1 | Phosphoribosylaminoimidazole carboxylase catalytic subunit [Acidithiobacillus caldus ATCC 51756]  >gb|EET28113.1| | 25.7 | 102 | Query 5 KIPFEAKV 12  KIPFEA V  Sbjct 30 KIPFEARV 37 |
| XP_002529173.1 | conserved hypothetical protein [Ricinus communis]  >gb|EEF33187.1| | 25.7 | 102 | Query 5 KIPFEAK 11  KIPFEAK  Sbjct 76 KIPFEAK 82 |
| ZP_01158734.1 | beta-galactosidase [Photobacterium sp. SKA34]  >gb|EAR57690.1| | 25.2 | 143 | Query 2 TKVKIPFEAK 11  T VK PFEAK  Sbjct 99 TNVKYPFEAK 108 |
| EEE25830.1 | signal recognition particle 54 kda protein, putative [Toxoplasma gondii GT1] | 25.2 | 143 | Query 1 ATKVKIPF 8  ATKV IPF  Sbjct 224 ATKVRIPF 231 |
| ZP_03130891.1 | hypothetical protein CfE428DRAFT_4057 [Chthoniobacter flavus Ellin428]  >gb|EDY18273.1| | 25.2 | 143 | Query 5 KIPFEAKV 12  KIPFE KV  Sbjct 493 KIPFEVKV 500 |
| CBZ55220.1 | Signal recognition particle GTPase, related [Neospora caninum Liverpool] | 25.2 | 143 | Query 1 ATKVKIPF 8  ATKV IPF  Sbjct 225 ATKVRIPF 232 |
| XP_002364522.1 | signal recognition particle 54 kda protein, putative [Toxoplasma gondii ME49]  >gb|EEA97381.1, EEE32999.1| | 25.2 | 143 | Query 1 ATKVKIPF 8  ATKV IPF  Sbjct 224 ATKVRIPF 231 |
| ZP_05852788.1 | peptidase, M20/M25/M40 family [Granulicatella elegans ATCC 700633]  >gb|EEW92316.1| | 25.2 | 144 | Query 2 TKVKIPFE 9  TK KIPFE  Sbjct 44 TKLKIPFE 51 |
| YP_699742.1 | PDZ domain-containing protein [Clostridium perfringens SM101]  >gb|ABG86295.1| | 25.2 | 144 | Query 4 VKI------PFEAKV 12  VKI PFEAKV  Sbjct 141 VKISSCDISPFEAKV 155 |
| ZP_06621505.1 | bacterial extracellular solute-binding protein [Turicibacter sanguinis PC909]  >ref|ZP_08168885.1|[Turicibacter sp. HGF1]  >gb|EFF64181.1|[Turicibacter sanguinis PC909]  >gb|EGC90806.1|[Turicibacter sp. HGF1] | 25.2 | 144 | Query 4 VKIPFEAK 11  VKIPFE K  Sbjct 49 VKIPFEEK 56 |
| ZP_02084191.1 | hypothetical protein CLOBOL_01715 [Clostridium bolteae ATCC BAA-613]  >gb|EDP17953.1| | 25.2 | 144 | Query 5 KIPFEAKV 12  KIPFE KV  Sbjct 19 KIPFEPKV 26 |
| XP_001802529.1 | hypothetical protein SNOG_12306 [Phaeosphaeria nodorum SN15]  >gb|EAT80119.1| | 25.2 | 144 | Query 2 TKVKIPFEA 10  TK+ IPFEA  Sbjct 41 TKIQIPFEA 49 |
| ZP_08261012.1 | GTP-sensing transcriptional pleiotropic repressor CodY [Gemella sanguinis M325]  >gb|EGF87972.1| | 25.2 | 144 | Query 1 ATKVKIPFE 9  ATKV IPF+  Sbjct 85 ATKVNIPFQ 93 |
| ZP_08258456.1 | GTP-sensing transcriptional pleiotropic repressor CodY [Gemella haemolysans M341]  >gb|EGF87278.1| | 25.2 | 144 | Query 1 ATKVKIPFE 9  ATKV IPF+  Sbjct 85 ATKVNIPFQ 93 |
| ZP_07953661.1 | GTP-sensing transcriptional pleiotropic repressor CodY [Gemella moribillum M424]  >gb|EFV36189.1| | 25.2 | 144 | Query 1 ATKVKIPFE 9  ATKV IPF+  Sbjct 85 ATKVNIPFQ 93 |
| XP_002978147.1 | hypothetical protein SELMODRAFT_417824 [Selaginella moellendorffii]  >gb|EFJ20804.1| | 25.2 | 144 | Query 3 KVKIPFEAKV 12  KVKIP EAK+  Sbjct 73 KVKIPAEAKI 82 |
| ZP_06192470.1 | hypothetical protein SOD_g01350 [Serratia odorifera 4Rx13]  >ref|YP_004498564.1|[Serratia sp. AS12]  >ref|YP_004503516.1|[Serratia sp. AS9]  >gb|EFA14949.1|[Serratia odorifera 4Rx13] >gb|AEF43255.1|[Serratia sp. AS9]  >gb|AEF48207.1|[Serratia sp. AS12]  >gb|AEG25915.1|[Serratia sp. AS13] | 25.2 | 144 | Query 3 KVKIPFEAKV 12  KVKIP EAK+  Sbjct 73 KVKIPAEAKI 82 |
| YP_001476368.1 | OsmC family protein [Serratia proteamaculans 568]  >gb|ABV39240.1| | 25.2 | 144 | Query 3 KVKIPFEAKV 12  KVKIP EAK+  Sbjct 73 KVKIPAEAKI 82 |
| XP_647029.1 | UBP-type zinc finger-containing protein [Dictyostelium discoideum AX4]  >gb|EAL73025.1| | 24.8 | 203 | Query 2 TKVKIPFE 9  TKVK PFE  Sbjct 1896 TKVKFPFE 1903 |
| XP_003291703.1 | hypothetical protein DICPUDRAFT_156322 [Dictyostelium purpureum]  >gb|EGC31780.1| | 24.8 | 203 | Query 2 TKVKIPFE 9  TKVK PFE  Sbjct 1283 TKVKFPFE 1290 |
| XP_001021881.1 | Helicase conserved C-terminal domain containing protein [Tetrahymena thermophila]  >gb|EAS01636.1| | 24.8 | 203 | Query 5 KIPFEAKV 12  KIPFE KV  Sbjct 853 KIPFEDKV 860 |
| XP_001800175.1 | hypothetical protein SNOG_09889 [Phaeosphaeria nodorum SN15]  >gb|EAT83154.2| | 24.8 | 204 | Query 2 TKVKIPFE 9  TKVKI FE  Sbjct 473 TKVKIAFE 480 |
| CBX91483.1 | similar to phosphoribosylformylglycinamidine cyclo-ligase [Leptosphaeria maculans] | 24.8 | 204 | Query 2 TKVKIPFE 9  TKVKI FE  Sbjct 425 TKVKIAFE 432 |
| XP_003302301.1 | hypothetical protein PTT_14058 [Pyrenophora teres f. teres 0-1]  >gb|EFQ89587.1| | 24.8 | 204 | Query 2 TKVKIPFE 9  TKVKI FE  Sbjct 425 TKVKIAFE 432 |
| XP_001941001.1 | phosphoribosylformylglycinamidine cyclo-ligase [Pyrenophora tritici-repentis Pt-1C-BFP]  >gb|EDU43720.1| | 24.8 | 204 | Query 2 TKVKIPFE 9  TKVKI FE  Sbjct 425 TKVKIAFE 432 |
| XP_003206165.1 | PREDICTED: acid trehalase-like protein 1-like [Meleagris gallopavo] | 24.8 | 204 | Query 1 ATKVKIPFEA 10  A KVK+PF+A  Sbjct 469 AKKVKVPFDA 478 |
| XP_420923.2 | PREDICTED: hypothetical protein [Gallus gallus] | 24.8 | 204 | Query 1 ATKVKIPFEA 10  A KVK+PF+A  Sbjct 471 AKKVKVPFDA 480 |
| ZP_01050345.1 | DNA ligase (NAD(+)) [Dokdonia donghaensis MED134]  >gb|EAQ38744.1| | 24.8 | 204 | Query 4 VKIPFEAKV 12  VKIPFE KV  Sbjct 498 VKIPFE-KV 505 |
| EGG23307.1 | inositol 5-phosphatase 2 [Dictyostelium fasciculatum] | 24.8 | 204 | Query 2 TKVKIPFE-AK 11  T VKIPFE AK  Sbjct 290 TIVKIPFELAK 300 |
| YP_004010317.1 | gp20 portal head vertex protein [Acinetobacter phage Acj9]  >gb|ADG60080.1| | 24.8 | 204 | Query 2 TKVKIPFEAKV 12  TKVKIP+ A V  Sbjct 223 TKVKIPYTAMV 233 |
| XP_002260372.1 | signal recognition particle [Plasmodium knowlesi strain H]  >emb|CAQ41639.1| | 24.8 | 204 | Query 1 ATKVKIPF 8  A KVKIPF  Sbjct 152 AAKVKIPF 159 |
| XP_001615888.1 | signal recognition particle 54 kDa protein [Plasmodium vivax SaI-1]  >gb|EDL46161.1| | 24.8 | 204 | Query 1 ATKVKIPF 8  A KVKIPF  Sbjct 152 AAKVKIPF 159 |
| XP_001348651.1 | signal recognition particle SRP54, putative [Plasmodium falciparum 3D7]  >gb|AAN37090.1|[Plasmodium falciparum 3D7]  >gb|AEA07718.1|[Plasmodium falciparum] | 24.8 | 204 | Query 1 ATKVKIPF 8  A KVKIPF  Sbjct 152 AAKVKIPF 159 |
| XP_678308.1 | signal recognition particle 54 kDa protein [Plasmodium berghei strain ANKA]  >emb|CAH96838.1| signal recognition particle 54 kDa protein, putative [Plasmodium berghei] | 24.8 | 204 | Query 1 ATKVKIPF 8  A KVKIPF  Sbjct 152 AAKVKIPF 159 |
| XP_726974.1 | signal recognition particle protein SRP54 [Plasmodium yoelii yoelii str. 17XNL]  >gb|EAA18539.1| | 24.8 | 204 | Query 1 ATKVKIPF 8  A KVKIPF  Sbjct 152 AAKVKIPF 159 |
| XP_001610843.1 | signal recognition particle SRP54 protein [Babesia bovis T2Bo]  >gb|EDO07275.1| | 24.8 | 204 | Query 1 ATKVKIPF 8  A KVKIPF  Sbjct 152 AAKVKIPF 159 |
| ADO76945.1 | DNA photolyase FAD-binding protein [Halanaerobium praevalens DSM 2228] | 24.8 | 204 | Query 1 ATKVKIPF 8  A KVKIPF  Sbjct 130 AAKVKIPF 137 |
| YP_002728405.1 | sodium symporter, ABC superfamily [Sulfurihydrogenibium azorense Az-Fu1]  >gb|ACN98552.1| | 24.8 | 204 | Query 4 VKIPFEAKV 12  V IPFE KV  Sbjct 2 VNIPFEPKV 10 |
| YP_004450453.1 | amidohydrolase [Haliscomenobacter hydrossis DSM 1100]  >gb|AEE53580.1| | 24.8 | 204 | Query 4 VKIPFEAKV 12  VKIPF KV  Sbjct 112 VKIPFASKV 120 |
| YP_003560579.1 | DNA replication and repair protein RecF [Bacillus megaterium QM B1551]  >ref|YP_003595321.1|[Bacillus megaterium DSM 319]  >gb|ADE67145.1|[Bacillus megaterium QM B1551]  >gb|ADF36971.1|[Bacillus megaterium DSM 319] | 24.8 | 204 | Query 2 TKVKIPFEAKV 12  TK IPFE KV  Sbjct 15 TKTTIPFENKV 25 |
| YP_267496.1 | AcrA/E family efflux transporter MFP subunit [Colwellia psychrerythraea 34H]  >gb|AAZ26934.1| | 24.8 | 204 | Query 2 TKVKI-PFE 9  TKVKI PFE  Sbjct 33 TKVKISPFE 41 |
| ZP_03131720.1 | conserved hypothetical protein [Chthoniobacter flavus Ellin428]  >gb|EDY17589.1| | 24.8 | 204 | Query 2 TKVKIPFE 9  T VKIPFE  Sbjct 2 TTVKIPFE 9 |
| ZP_04777250.1 | GTP-sensing transcriptional pleiotropic repressor CodY [Gemella haemolysans ATCC 10379]  >gb|EER67958.1| | 24.8 | 204 | Query 1 ATKVKIPF 8  ATKV IPF  Sbjct 85 ATKVNIPF 92 |
| YP_003716865.1 | hypothetical protein CA2559_10598 [Croceibacter atlanticus HTCC2559]  >gb|EAP86478.1| | 24.8 | 204 | Query 2 TK--VKIP-FEAKV 12  TK KIP FEAKV  Sbjct 150 TKNDAKIPVFEAKV 163 |
| BAD36837.1 | hypothetical protein [Thermus sp. TK10] | 24.8 | 204 | Query 1 ATKVK---IPFEAK 11  ATKV PFEAK  Sbjct 110 ATKVRQKDFPFEAK 123 |

Note: * Proteins that show lower homologue scores are not listed here. Homology analysis results obtained with other peptides are not shown here either, since it will be more convenient for the readers to do blastp analysis for themselves.
