# Supplementary material for: Phage Display against Corneal Epithelial Cells Produced Bioactive Peptides That Inhibit Aspergillus Adhesion to the Corneas
Source: PLoS One. 2012 Mar 12;7(3):e33578. doi: 10.1371/journal.pone.0033578 (PMC3299800; doi:10.1371/journal.pone.0033578)
Supplement: Table S3 — Enriched gene functions and pathways all proteins of Aspergillus fumigatus that show high homology with 14 peptides. The first 100 homologue sequences that gave the highest homologue scores for each of the 14 peptides were combined and subjected to DAVID analysis. (DOC) [file pone.0033578.s004.doc]

**Table S3**. Enriched gene functions and pathways for homologue proteins of Aspergillus fumigatus corresponding to 14 peptides

| Terms | Count | % | EASE P Value | Fold Enrichment |
| --- | --- | --- | --- | --- |
| Gene functions | | | | |
| ATP-binding | 54 | 6.667 | 1.63E-05 | 1.831 |
| Nucleotide-binding | 58 | 7.160 | 1.70E-04 | 1.644 |
| Phosphopantetheine | 9 | 1.111 | 3.72E-04 | 4.657 |
| Transcription | 28 | 3.457 | 5.59E-04 | 2.020 |
| Transcription regulation | 26 | 3.210 | 8.18E-04 | 2.036 |
| Aminoacyl-tRNA synthetase | 9 | 1.111 | 0.012 | 2.819 |
| Nucleus | 42 | 5.185 | 0.021 | 1.408 |
| Helicase | 14 | 1.728 | 0.026 | 1.938 |
| Transit peptide | 6 | 0.741 | 0.039 | 3.105 |
| Ligase | 21 | 2.593 | 0.039 | 1.592 |
| Chromatin regulator | 6 | 0.741 | 0.046 | 2.976 |
| KEGG Pathways | | | | |
| Aminoacyl-tRNA-biosynthesis | 9 | 1.111 | 0.014 | 2.716 |
| Meiosis | 10 | 1.234 | 0.034 | 2.180 |

For each peptide, the first 100 homologue sequences, either biologically characterized protein or putative protein, that gave the highest homologue scores were retrieved and combined for all 14 peptides and subjected to DAVID analysis. In Aspergillus fumigatus, 810 valid DAVID GI were obtained corresponding to the 1400 sequences and all of them fell into SP_PIR_KEYWORDS groups in FUNCTIONAL CATEGORIES. The 11 categories with the EASE value below 0.05 were regarded enriched in the homologues sequences. Among all 810 DAVID GI, 182 (15.5%) were recorded in the KEGG_Pathway category, and the two enriched pathways were Aminoacyl-tRNA-biosynthesis and meiosis, respectively.
